# The immunostimulatory role of an *Enterococcus-*dominated gut microbiota in host protection against bacterial and fungal pathogens in *Galleria mellonella* larvae

**DOI:** 10.3389/finsc.2023.1260333

**Published:** 2023-10-26

**Authors:** Jennifer Upfold, Agnès Rejasse, Christina Nielsen-Leroux, Annette Bruun Jensen, Vincent Sanchis-Borja

**Affiliations:** ^1^ Université Paris-Saclay, INRAE, AgroParisTech, Micalis Institute, Jouy-en-Josas, France; ^2^ Department of Plant and Environmental Sciences, University of Copenhagen, Frederiksberg, Denmark

**Keywords:** *Bacillus thuringiensis*, *Metarhizium robertsii*, gene expression, gut microbiota, tripartite interactions, axenic

## Abstract

Understanding the intricate interplay between the gut microbiota and the immune response in insects is crucial, given its diverse impact on the pathogenesis of various microbial species. The microbiota’s modulation of the host immune system is one such mechanism, although its complete impact on immune responses remains elusive. This study investigated the tripartite interaction between the gut microbiota, pathogens, and the host’s response in *Galleria mellonella* larvae reared under axenic (sterile) and conventional (non-sterile) conditions. The influence of the microbiota on host fitness during infections was evaluated via two different routes: oral infection induced by *Bacillus thuringiensis* subsp. *galleriae* (*Btg*), and topical infection induced by *Metarhizium robertsii* (*Mr*). We observed that larvae without a microbiota can successfully fulfill their life cycle, albeit with more variation in their developmental time. We subsequently performed survival assays on final-instar larvae, using the median lethal dose (LD_50_) of *Btg* and *Mr*. Our findings indicated that axenic larvae were more vulnerable to an oral infection of *Btg*; specifically, a dose that was calculated to be half-lethal for the conventional group resulted in a 90%–100% mortality rate in the axenic group. Through a dual-analysis experimental design, we could identify the status of the gut microbiota using 16S rRNA sequencing and assess the level of immune-related gene expression in the same group of larvae at basal conditions and during infection. This analysis revealed that the microbiota of our conventionally reared population was dominated entirely by four *Enterococcus* species, and these species potentially stimulated the immune response in the gut, due to the increased basal expression of two antimicrobial peptides (AMPs)—gallerimycin and gloverin—in the conventional larvae compared with the axenic larvae. Furthermore, *Enterococcus mundtii*, isolated from the gut of conventional larvae, showed inhibition activity against *Btg in vitro*. Lastly, other immune effectors, namely, phenoloxidase activity in the hemolymph and total reactive oxygen/nitrogen species (ROS/RNS) in the gut, were tested to further investigate the extent of the stimulation of the microbiota on the immune response. These findings highlight the immune-modulatory role of the *Enterococcus-*dominated gut microbiota, an increasingly reported microbiota assemblage of laboratory populations of Lepidoptera, and its influence on the host’s response to oral and topical infections.

## 1 Introduction

Insects are essential contributors to the global ecosystem, performing important ecosystem services in agriculture such as pollination and biological control; they are heavily used for research purposes, and, more recently, for sustainable food production (1–3). Moreover, their potential to be produced in large-scale production facilities holds promise for addressing various environmental and nutritional challenges confronting our world. However, inherent risks arise when handling large numbers of animals such as disease outbreaks, which commonly occur among mass-reared animals. This could create consequences for public health, as well as raise concerns for animal welfare, if diseases are not adequately managed (4, 5).

Insects have evolved innate immune systems that allow them to defend themselves against invading pathogens and subsequent diseases. They possess an innate immune response that can be elicited via cellular or humoral means (6). Their cellular immune responses are mediated by hemocytes and include phagocytosis, nodulation, and encapsulation, which are capable of fighting against a large spectrum of pathogens (7). The humoral immune response involves processes such as melanization via the activation of the phenoloxidase system, the secretion of molecules such as antimicrobial peptides (AMPs), and the production of reactive oxygen/nitrogen species (ROS/RNS) (6, 8, 9). The gut of insects, which harbors a complex ecosystem of microorganisms known as the microbiota, is also recognized as playing a crucial role in maintaining the overall health of its host by modulating various activities such as nutrient absorption, digestion, and immune system development, among other important biological processes (10–12). Therefore, understanding the role of the insect gut microbiota and its interactions with its host’s immune system and pathogens is essential for both insect control and insect health.

The nature and dynamic of the microbiota–immune response interaction can vary greatly across different insect groups, in part due to the differences in the bacterial species comprising the gut microbiota, but also depending on factors such as diet, habitat, life stage, sex, and social interactions, resulting in differences in the scope of benefits provided to the host (10). The gut microbiome, in a balanced state, can maintain a symbiotic relationship; however, the relationship exists on a continuum that can shift toward neutrality or pathogenicity. When functioning at its best, this immune system–microbiota partnership enables protective responses against pathogens, while avoiding excessive immune activation that can harm host tissues or disrupt the composition and diversity of the symbiotic gut microbiota, thus preventing opportunistic infections by pathogenic bacteria or fungi (6, 13–15).

In some cases, the microbiota may actually facilitate pathogen infections, particularly when there are disruptions to the community caused by dysbiosis. This can result in an elevated risk of septicemia when bacteria breach the gut barrier and enter the hemocoel (16, 17). Although the role of the gut microbiota in enhancing host resistance to infections is increasingly studied, the current knowledge of its role and contribution to host tolerance by different pathogens is still limited and fragmented. Although the gut microbiota has been observed to contribute to the defense against oral infections by invading pathogens in a wide range of animals, from invertebrates to vertebrates, less is known about its role in defending against topical pathogens (18, 19). The gut can be an important site of immune resistance to a variety of pathogens that enter not only orally but also topically, as is typically observed in entomopathogenic fungi, which enter their host primarily through the cuticle. Therefore, this interaction between insect gut microbiota and fungi needs to be further explored.

In this study, our aim was to investigate the partnership between the gut microbiota and the immune response during single infections by pathogens that infect *Galleria mellonella* (L.) (Lepidoptera: Pyralidae) larvae through different routes. *G. mellonella* serves as a widely utilized model organism for studying host–pathogen interactions, given its similarities to the mammalian immune system and its tolerance of temperatures ≥ 37°C. As a result, there is an increasing amount of published information available on its immune system (6, 20, 21). In addition, the microbiota of *G. mellonella* has been the subject of growing research interest, particularly due to its possible ability to break down polyethylene (22–24). To gain insights into the role of the microbiota in protecting the host against infectious agents, we specifically focused on both conventional and axenic (microbiota-free) larvae (25, 26).

To test the immune response via oral infection, we used *Bacillus thuringiensis* subsp. *galleriae* 69-6 (Btg), a pathogenic strain known to infect *G. mellonella* (27, 28). *B. thuringiensis* is a Gram-positive, spore-forming bacteria found in soil environments, which produces cry and cyt δ-endotoxins with entomopathogenic properties. When ingested, the toxins solubilize and interact with specific receptors on the midgut epithelial cells, leading to gut epithelium breakdown and potential septicemia (29, 30). For topical infection, we utilized *Metarhizium robertsii*, an entomopathogenic fungus that can infect various insect species, including *G. mellonella.* The fungus penetrates the host from the cuticle surface via the formation of germ tubes and cuticle-degrading enzymes, where it then grows rapidly in the hemocoel and destroys important structures of the host (31). These two pathogens produce spores or conidia that can contaminate many environments, from food to water or air, and, therefore, have the potential to be introduced into insect farms through contaminated feed sources, equipment, or even via simple airborne transmission (5).

Specifically, we evaluated the effects of exposure to these two naturally occurring pathogens, entering the host orally and topically, by analyzing the size and composition of the conventional larvae gut microbiota community. We further examined how the composition and diversity of the gut bacterial microbiota, as well as the absence of microbiota, affect the severity of infection, the immune response, and growth and development (*i.e.*, time to pupation, pupal duration, and adult duration). To achieve this, we used a combination of 16S rRNA gene amplicon sequencing to quantify the dominant taxa and subsequently evaluate the microbial dysbiosis provoked by the pathogens, and target qRT-PCR analysis of immune response genes in the gut to determine the impact of the gut composition on the expression of three target genes involved in the immune response. Our findings shed light on the complex interactions between the gut microbiota and host immunity and have implications for the development of novel strategies for controlling infectious diseases.

## 2 Materials and methods

### 2.1 Insect rearing

A laboratory population of *G. mellonella* L. (Lepidoptera: Pyralidae) larvae reared at the Micalis Institute of INRAE, Jouy en Josas, France, was used in the following study. The population was reared on a nutritionally rich, natural diet of beeswax and pollen (La Ruche Roannaise, France). The nutritional compounds in pollen included essential amino acids, carbohydrates, a few lipids, and some vitamins and bioelements; the wax served as a source of fatty acids and other complex long-chain carbohydrates that *G. mellonella* can metabolize (32). The rearing chamber was set at 28°C with a 12-hour photoperiod.

#### 2.1.1 Preparation of axenic larvae

The production and manipulation of axenic larvae, defined as having an absence of bacteria in their gut microbiota, were done in sterile conditions. To produce the larvae, the materials and egg sheets were exposed to a 10-minute surface sterilization by ultraviolet (UV) light at 254 nm in a biosafety cabinet. The sterilized egg sheets were then placed into an autoclaved sterile glass jar that was sealed with a fine mesh (with a hole diameter of 0.596 mm) and covered with natural-fiber cotton wool. The conventional and axenic larvae were fed the same diet; however, the diet for axenic larvae was sterilized via gamma irradiation (sterilization was carried out using cobalt-60 at the intensity of 25 kGy; SAFE, Augy, France). The axenic status was verified on five larvae by plating a suspension of the crushed and homogenized larvae on BHI (brain heart infusion) agar. Further verification by PCR targeting the bacterial 16S rRNA gene using the V3–V4 region (forward: 5′-TACGGGAGGCAGCAG-3′; and reverse: 5′-CCAGGGTATCTAATCC-3′; 33, 34) was conducted on DNA extracted from dissected guts (**Supplementary Figure S1**).

### 2.2 Biological assay

The various life stages of the axenic and conventional larvae were monitored from the egg to the adult stage to determine if there were any differences in the biological parameters of larvae without a microbiota. The eggs of the conventional and axenic adults were collected after 2 days (approximately 250 eggs per sheet) and placed into sterile glass jars supplemented with food. A natural sterilized diet was provided to the axenic group, and a non-treated natural diet was provided to the conventional group. This was an important step to ensure that the conventional larvae would have a colonized gut microbiota. The larvae were left for approximately 2 weeks until they were large enough to handle (40 mg). They were then individually placed in 24-well plates and provided with a nutritious artificial sterilized wheat-based diet (35) (**Supplementary Ta** **ble S1**), where they were monitored until pupation. The pupae were then moved to medicine cups covered in cotton wool where the adults would eclose and remain until death. The adults did not feed due to degenerative mouthparts and were therefore not provided with food or water (36).

### 2.3 Pathogen infection assays

#### 2.3.1 Oral infection—*Bacillus thuringiensis*

The bacterium *Bacillus thuringiensis* subsp. *galleriae* 69-6 (*Btg*) obtained from Dr. Ekaterina V. Grizanova, Department of Plant Protection, Novosibirsk State Agrarian University, was used to induce the immune response by oral infection. The strain was transformed with pHT315ΩP*aphA3-gfp* to express GFP (green fluorescent protein). The *Btg* cells were grown with shaking at 30°C for 96 h in HCT (0.7% casein hydrolysate, 0.5% tryptone, 0.68% KH_2_PO_4_, 0.012% MgSO_4_ • 7H_2_O, 0.00022% MnSO_4_ • 4H_2_O, 0.0014% ZnSO_4_ • 7H_2_O, 0.008% ferric ammonium citrate, and 0.018% CaCl_2_ • 4H_2_O, pH 7.2) + 0.5% glucose medium. After 72 h, sporulated cells and crystals were subjected to three washing (in 1M NaCl) and centrifugation (4°C, 5,000 G) cycles. After the final centrifugation, the spores/crystals were resuspended in double-distilled water (ddH_2_O). The oral infection was undertaken on ± 250-mg larvae by force feeding with a cannula hypodermic needle (30 gauge × 25 mm) and a 1-mL syringe (Terumo Corporation). A spore and crystalline toxin mixture of 10 µL was administered at a half-lethal dose [5.2 × 10^5^ colony-forming units (CFU) in 10 µl], determined in previous assays using only conventional larvae. The uninfected control larvae were force fed with 10 µL of ddH_2_O. The inoculated larvae were then placed in Petri dishes (60 mm × 15 mm) and kept in a 30°C incubator to monitor the infection over 96 h. For the mortality assays, a total of 60 larvae were infected over three replicates.

#### 2.3.2 Topical infection—*Metarhizium robertsii*

The fungus *Metarhizium robertsii* KVL 00-89 (*Mr*) was obtained from the culture collection at the Section for Organismal Biology at the Department of Plant and Environmental Sciences of the University of Copenhagen, Copenhagen, Denmark. To induce the immune response by a topical infection, *Mr* was grown on Sabouraud dextrose agar (SDAY) plates (16.25 g of SDA (Sigma-Aldrich), 2.5 g of yeast extract, and 11.25 g of agar in 1 L distilled water) at 30°C. The conidia were harvested after 3 weeks and a Fuchs–Rosenthal counting chamber (Hausser Scientific) was used to determine the concentration. The conidial suspensions of *Mr* were prepared at 1 × 10^7^ conidia in a 3-mL solution of 0.05% Triton X. The larvae were completely submerged in the conidial suspension for 30 s, and then placed in a sterile Petri dish lined with filter paper. All conidial suspensions had a germination rate of between 95% and 100%. The control larvae were submerged in a solution of 0.05% Triton X. The larvae were then placed in a 30°C incubator to monitor the infection up to 240 h, or until the surviving larvae pupated. For the mortality assays, a total of 60 larvae were infected over three replicates.

### 2.4 Clearance assay of *Btg*

To investigate the rate of clearance of *Btg* in the gut of the axenic and conventional larvae, 30 larvae were infected at a half-lethal dose (calculated in the conventional larvae). Every 24 h, the CFU counts of *Btg* were monitored by plating the dissected guts of three surviving larvae (individually) on to lysogeny broth agar (LBA). The guts were dissected in sterile conditions and homogenized in 500 µL of ddH_2_O. This was monitored for up to 96 h.

### 2.5 Dual-action analysis of immune response genes in the gut tissue and microbiota assessment

To investigate the relationship between the gut microbiota and immune response, surviving larvae infected by either *Btg* or *Mr* were collected at specific time points, representing early and late stages of infection. For the *Btg* treatment, larvae were collected at 20 h and 40 h post infection; for *Mr*, the larvae were collected at 20 h and 96 h, as *Mr* takes a longer time to achieve mortality in its host. The collected larvae were subjected to a 30-min cooling period on ice, followed by surface sterilization using ethanol and subsequent gut dissection. The gut was homogenized in 200 μL of ddH_2_O using a sterile pestle prior to splitting the contents into two Eppendorf tubes, with one half intended for a qRT-PCR analysis of immune response genes and the other for a 16S rRNA microbiota assessment. Half guts from two larvae were mixed, with each biological replicate comprising two larvae. Therefore, we could assess the changes occurring in the microbiota and the expression of immune response genes in the same group of larvae (**Supplementary Figure S2**).

#### 2.5.1 DNA extraction of gut samples and axenic verification

The DNA from gut samples was extracted using the DNeasy® PowerSoil® Pro Kit (Qiagen) in accordance with the manufacturer’s instructions, with slight modifications. The samples were incubated at 65°C with shaking for 10 minutes prior to being lysed in a FastPrep-24™ (MP Biomedicals) for 2 × 40 s at 4.0 M/S with a 5-min break at 4°C. The quality of the extractions was checked on a NanoDrop™ spectrophotometer (Thermo Fisher Scientific) and a 1.2% agarose gel. For axenic samples, a PCR targeting the bacterial 16S rRNA gene using the V3–V4 region (forward: 5′-TACGGGAGGCAGCAG-3′; and reverse: 5′-CCAGGGTATCTAATCC-3′; 33, 34) was conducted to verify axenic status (**Supplementary Figure S1B**).

#### 2.5.2 16S rRNA gene sequencing

The DNA extractions of the conventional larvae (with controls including a Zymobiomics™ microbiome standard and two axenic samples) were further processed by Eurofins Genomics (France). This included PCR reactions, library preparation, and sequencing, which was performed on an Illumina MiSeq™ (2 × 300 bp). The resulting sequences were further processed on RStudio using the “DADA2” package to obtain an amplicon sequence variant (ASV) table, which identifies fine-scale variations compared with the more traditional operational taxonomic unit (OTU) table (37). Based on the inspection of the quality profiles and error rates, some modifications were made to the proposed functions by the DADA2 workflow and package. Taxonomic affiliations were performed using the SILVA2 database developed by the Leibniz Institute (38), and NCBI BLAST was used on ASV sequences to further identify to species level. The relative abundance plots and alpha- and beta-diversity metrices were generated using the R package “Phyloseq” (39). The permutational multivariate analysis of variance (PERMANOVA) was conducted on beta-diversity estimates with significance at < 0.05 using the adonis() function from the R “vegan” package (40) (see **Supplementary Materials and Methods**).

#### 2.5.3 RNA extraction, cDNA synthesis, and qRT-PCR of immune response genes in the gut

RNA extractions using TRIzol™ reagent (Invitrogen) were performed on the other half of the dissected and homogenized gut samples. The quality and quantity of the extractions were estimated on a NanoDrop spectrophotometer and via gel electrophoresis. The RNA quality was verified prior to being transformed into cDNA with SuperScript™ IV VILO™ master mix (Thermo Fisher Scientific), in accordance with the manufacturer’s instructions. To investigate the role of the host gut bacteria on the expression of immune response genes in the gut tissue, we performed qRT-PCR on four immune-related genes (**Supplementary Tables S2, S3**): two antimicrobial peptide genes (gallerimycin and gloverin), a lysozyme-like gene (lysozyme), and an insect metalloproteinase inhibitor (*IMPI*). The relative expression of each target gene was normalized to a housekeeping gene that is known for its stable expression across different experimental conditions: 18S rRNA (Ivan M. 41). Therefore, the data are presented as a ratio over 18S, taking into account the reaction efficiencies of the PCR. Subsequently, a heatmap of the fold change (log2) in gene expression from basal to infected conditions by either *Btg* or *Mr* is presented. The significance was determined for the difference in the fold change in expression of each gene and the time point between the conventional and axenic larvae, by one-way ANOVA on log2-transformed values.

### 2.6 Inhibition assay

The *Enterococcus mundtii* strain isolated from the conventional larvae gut (see **Supplementary Material and Methods**, **Supplementary F** **igure S3**) was screened using the agar spot-on lawn technique (42), as conducted by Grau et al. (43) along with minor modifications that are further described. First, overnight cultures of the *E. mundtii* strain was grown in a MRS (deMan, Rogosa, and Sharpe) medium at 30°C without agitation. Approximately 7 µL was then spotted onto 0.7% MRS agar plates and incubated for a further 24 h under aerobic conditions at 30°C. After the 24-hour incubation, the plates were exposed to UV light at 254 nm for 25 min in a sterile biosafety cabinet. This action was taken to kill the *E. mundtii* colonies, resulting in any inhibitory affect coming from only the bacteriocins already secreted into the agar. An overnight culture of the indicator bacteria, *Bacillus thuringiensis* subsp. 69-6, was mixed with 1% LBA. Approximately 10 mL of the indicator bacteria was poured over the previously *E. mundtii* “spotted” and UV-exposed plates. Once solidified, the plates were incubated for a further 48 h at 30°C under aerobic conditions. The plates were then assessed for inhibition zones, and subsequent zones were measured using a ruler (**Supplementary Figure S4**).

### 2.7 Phenoloxidase activity assay

The phenoloxidase (PO) activity in the hemolymph of larvae was measured using a technique described by Valadez-Lira et al. (44), who adapted a method from Harizanova et al. (45). Slight modifications were made as further described. Approximately 10µL of hemolymph was individually collected via puncturing the fourth proleg with a sterile microneedle, and directly pipetting the bleeding hemolymph into an Eppendorf tube containing 190 µL of ice-cold sterile 1 × phosphate-buffered saline (PBS). This was done at 20 h and 40 h for the *Btg* infection, and at 20 h and 96 h for the *Mr* infection. The homogenates were frozen at –80°C for at least 24 h, which allowed the hemocytes to lyze and release the inner-cell plasma. The enzymatic assay was then conducted on thawed (on ice) and centrifugated (8,000 g, 4°C, 5 min) samples by preparing a flat-bottomed 96-well plate with 30 µL of the sample, mixed with 100 µL of 16 mM L-DOPA (**Supplementary Figure S5**) (3,4,dihyroxy-L-phenylalanine). As a negative control, PBS was used. The plates were read immediately at 490 nm every 15 s (with 3 s of shaking between reads) at 30°C on a photospectrometer (Spectra Max 190) using SoftMax™ Pro 7.1 software. The PO activity was measured from the slope (*V*_max_) of the reaction curve in its linear phase. The samples were run in duplicate for a total of five individual larvae tested per time point and treatment, with three independent experiments for the *Btg* infection and two independent experiments for the *Mr* infection.

### 2.8 ROS/RNS assay

Reactive oxygen species (ROS) and reactive nitrogen species (RNS) are the total free radicals generated during cellular processes and immune responses. Measuring total ROS/RNS helps evaluate oxidative stress and its impact on host fitness during infection. To determine their levels, the total ROS/RNS was quantified in the surviving larvae gut during oral infection by *Btg* and topical infection by *Mr*. The guts from three surviving larvae were dissected, pooled, and homogenized in sterile PBS at 20 h and 40 h for *Btg* infection, and at 20 h and 96 h for *Mr* infection. Three samples were prepared for a total of nine larvae per time point and treatment (three pooled guts in three separate samples). The samples were processed using the OxiSelect™ *In Vitro* ROS/RNS Assay Kit (Cell Biolabs, USA) following the manufacturer’s instructions. Approximately 100-fold diluted samples and kit components were incubated on a 96-well black-bottom plate for 20 min at room temperature. Fluorescence was measured on an Infinite® M200 Pro microplate spectrofluorometer (Tecan, USA), and the results were obtained using a predetermined dichlorofluorescein (DCF) standard curve linear regression equation.

### 2.9 Data analysis

For mortality assays, the log-rank test was performed with Holm–Šídák adjustment on RStudio using the survival package (RStudio™ 2010). All other data were analyzed using GraphPad Prism v.9.3 (GraphPad Software Inc., USA). The data were always checked for normality using the Shapiro–Wilk *W*-test prior to determining the statistical tests. Based on the normality statistic, the unpaired *t*-test was performed at each time point of the clearance assay as well as for the various biological parameters between axenic and conventional larvae.

## 3 Results

To investigate the relationship between the gut microbiota and the host’s tolerance to oral and topical pathogens in *G. mellonella*, axenic larvae were generated via UV sterilization of the eggs followed by sterile rearing on a germ-free natural diet. The axenic status of the larvae was verified by dissecting the whole gut and performing CFU counts on BHI agar plates, as well as by PCR amplification of the V3–V4 region of the 16S gene. As a final confirmation, two dissected gut samples from the axenic larvae were included in the Illumina sequencing. No cultivable bacteria were present in the axenic larvae gut, no amplification of the V3–V4 region was observed, and no reads were generated from the Illumina sequencing (**Supplementary Figure S1**). In comparison, the conventional larva gut-maintained bacteria were at a concentration of between 1 × 10^7^ and 10^8^ CFU/mL per larva.

### 3.1 Axenic group experienced a longer and more variable larval stage

**Figure 1:**
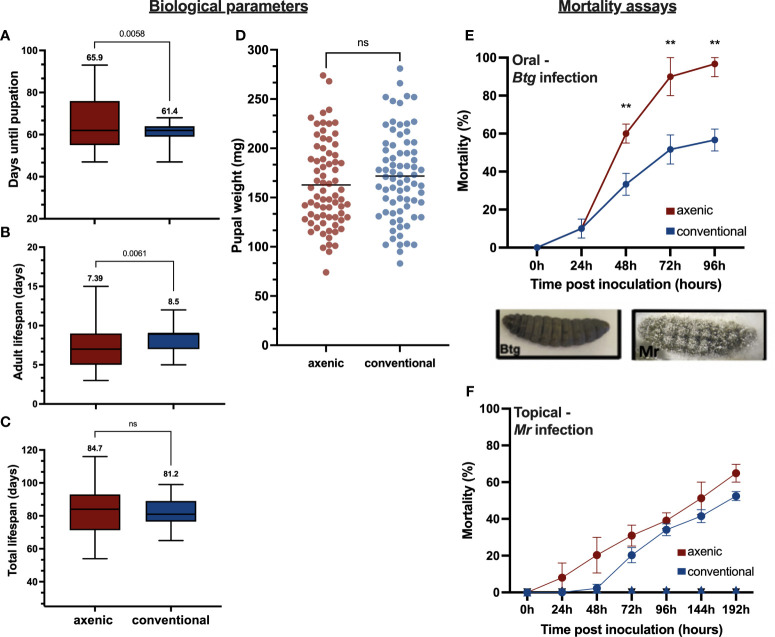
Various life history traits measured between the axenic and conventional larvae (n = 75 per group), including **(A)** the number of days from hatching until pupation, **(B)** the length of the adult life span, **(C)** the total life span, and **(D)** the pupal weight. The percentage mortality for the axenic and conventional larvae was calculated for **(E)** *Btg* infected larvae by orally force-feeding the LD50 (2 × 106 in 10 µL of a spore crystal mix) and recorded over 96 hours (three replicates, with n = 60); **(F)** for *Mr* infected larvae by topically submerging larvae into conidial suspension (1 x 107 conidia in 3mL TritonX 0.05%) and recorded over 192 hours (three replicates, with n = 90). No mortality was recorded for the controls. The different phenotypes of the cadavers post infection by *Btg* and *Mr* are also displayed. *Btg*-infected larvae are completely melanized upon death, whereas the *Mr*-infected larvae have fungal hyphae growing out of the cadaver at 72 h post death. ns, not significant.

We observed some differences in the various life stages of the insect between axenic and conventional larvae. The larval developmental time, that is, from egg to pupation, was significantly longer in the axenic larvae, with an average of 65.9 days, than in the conventional larvae, which took on average 61.4 days (*p* = 0.0058) (**Figure 1A**). Six individuals from the conventional group and nine individuals from the axenic group did not emerge as adults. There was also a significant difference in the length of the adult life span (**Figure 1B**) (*p* = 0.0033). The mean average length for the conventional adults was 8.5 days compared with 7.39 days in the axenic group, albeit the axenic larvae experienced greater variation, with the shortest life span being 2.5 days and the longest being 15 days. Comparatively, the longest adult life span in the conventional group was 12.5 days and the shortest was 5 days. There was no significant difference in the total life span (**Figure 1C**) and pupal weights (**Figure 1D**).

### 3.2 Axenic larvae were more susceptible to orally inoculated *Btg*

Different rates of survival were observed between axenic and conventional larvae post-oral *Btg* infection (**Figure 1E**). The larvae reared axenically were more susceptible to a half-lethal dose of a *Btg* spore crystal preparation, with a significantly greater mortality from 40 h post infection (60%—χ^2^ > 9.7, df = 1; *p* = 0.002), which was maintained at 72 h (90%—χ^2^ > 15.2, df = 1; *p* = 0.0004) and 96 h (96.6%—χ^2^ > 11.7, df = 1; *p* = 0.0007). For the *Mr* topical infection, no significant difference was observed between the axenic and conventional larvae at a half-lethal dose (**Figure 1F**). However, slightly faster rates of mortality were observed in the axenic larvae in the first 72 h.

### 3.3 Conventional larvae experienced a faster rate of *Btg* clearance from the gut

**Figure 2:**
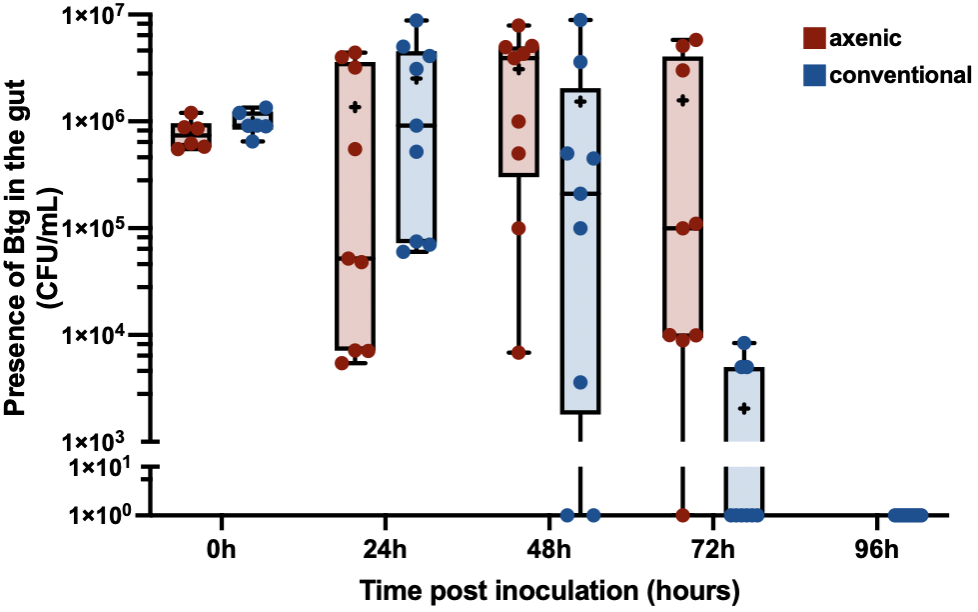
The clearance of *Btg* in the gut of surviving larvae over 96 hours: the dissected gut from surviving larvae post infection by *Btg* at 0, 24-, 48-, 72 and 96 hours was plated on LBA to count the resulting colony-forming units (CFU). By 72 h post infection the conventional larvae (in blue) were able to clear *Btg* to a sublethal concentration and, furthermore, completely clear the infection by 96 h. Comparatively, the number of spores in the axenic larvae (in red) remained high, even at 72 h, resulting in 100% mortality, and, therefore, no clearance data for the axenic group by 96 h.

To further investigate the difference in *Btg* pathogenicity between axenic and conventional larvae, an assay to assess the rate of clearance of *Btg* from the gut in surviving larvae was performed. Along with the different rate of clearance between the axenic and conventional larvae (**Figure 2**), we also observed considerable variation in the clearance between individual larvae. At 48 h post inoculation, some surviving conventional larvae were able to completely clear *Btg*, while the concentration remained above 5.2 × 10^3^ CFU/mL in axenic larvae. By 72 h, the conventional larvae cleared the spores to that of a sublethal level, and at 96 h all surviving larvae tested completely cleared the spores. However, at 72 h the average concentration of spores in the axenic group remained above the lethal concentration, and at 96 h all remaining larvae died.

### 3.4 The bacterial gut microbiota was dominated by *Enterococcus* spp.

**Figure 3:**
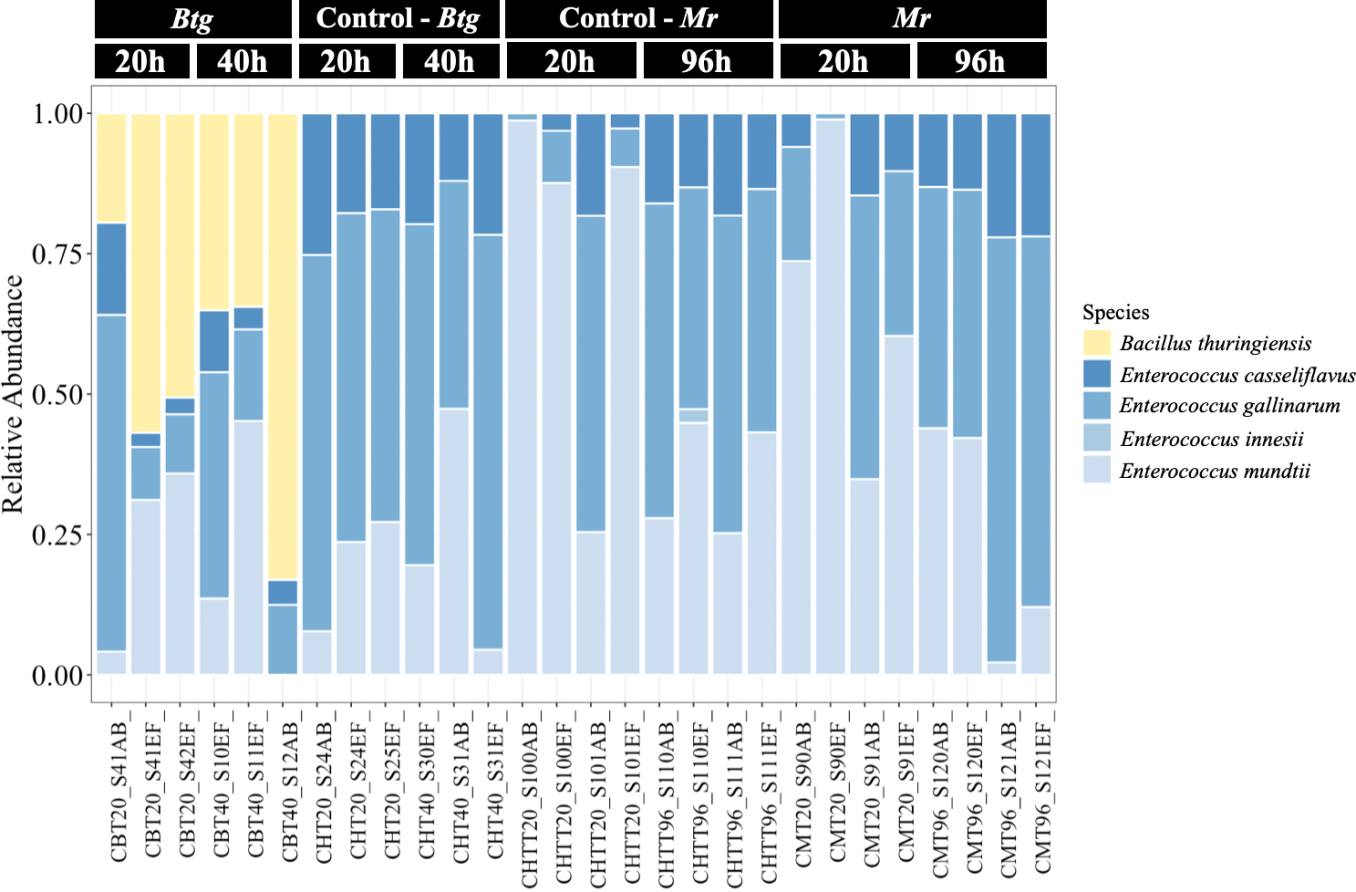
The relative abundances of the bacterial species in the gut of the conventional larvae at two time points for orally infected larvae with *Bacillus thuringiensis* subsp*. galleriae* 69-6 (*Btg)*, and topically infected with *Metarhizium robertsii* (*Mr*), as well as the controls (larvae force-fed sterile water for the *Btg* group and submerged in 0.05% Triton X for the *Mr* group). The plots display the proportion of reads post rarefied (35,000), normalized (per 100), and filtered (0.1/100).

We performed bacterial taxonomic gene sequencing on the bacterial gut microbiota of 56 conventional larvae to assess the community structure during infection by oral and topical pathogens, in order to infer how changes to the community may be affecting host susceptibility during pathogenesis. Two axenic samples were also sequenced but generated no reads, verifying their sterility. The sequencing of the conventional larvae revealed that the gut microbiota of our *G. mellonella* population was dominated by only one bacterial genus: *Enterococcus.* The ASVs showed strong similarity with four different species—*Enterococcus casseliflavus, Enterococcus gallinarum, Enterococcus innesii*, and *E. mundtii* (**Figure 3**)—as determined using the SILVA16S and BLAST databases (**Supplementary Table S4**). We found variation in the proportion of the four species across different cohorts of larvae; however, *E. casseliflavus* and *E. innessi* were consistently in a minority compared with *E. gallinarum* and *E. mundtii*, which dominated the community. When larvae were infected with a median lethal dose (LD_50_) of *Btg*, its presence was recovered in all infected samples at 20 h post infection (*n* = 6 larvae), with > 50% dominance of the overall community in two of the three samples. At 40 h post infection, the presence of *Btg* again varied across the samples (*n* = 6 larvae), with two samples showing a reduction in *Btg* to that of what was observed at 20 h, and one sample containing > 80% *Btg* reads (**Supplementary Fi** **gure S6**). *Mr* did not have an effect on the bacterial community structure at either 20 h (*n* = 8 larvae) or 96 h (*n* = 8 larvae), with similar relative abundances observed in the controls (*n* = 8 larvae at each time point). The alpha-diversity matrices also showed no significant differences between *Btg-* or *Mr-*infected samples and their control samples in both the Observed and the Simpson Index (**Supplementary Figure S7**). For the beta-diversity analysis, all ASVs assigned to *Btg* were subsetted and discarded using the “subset_taxa” function on phyloseq. This analysis was done to assess the impact of *Btg* on the rest of the community without the influence of the introduced *Btg* affecting the results (**Supplementary Figure S8**). The principal component analysis (PCoA) of the data subsets revealed a small grouping of three *Btg*-infected samples: two from 20 h and one from 40 h post infection. However, further analysis by PERMANOVA revealed no significance between the time or treatments (**Supplementary Ta** **ble S5**). This suggests that the microbiota community remained relatively stable (i.e., no shifts at the genus or species level) during infection as compared with the uninfected microbiota community. Finally, our analysis highlights the inter-individual variation of the proportion of *Btg* (in *Btg-*infected samples) and the *Enterococcus* spp. occurring in the microbiota community between larvae, which is an important aspect to consider and is often lost in group-level analyses.

### 3.5 Isolated *E. mundtii* from the conventional larvae gut microbiota inhibited *Btg in vitro*

**Figure 4:**
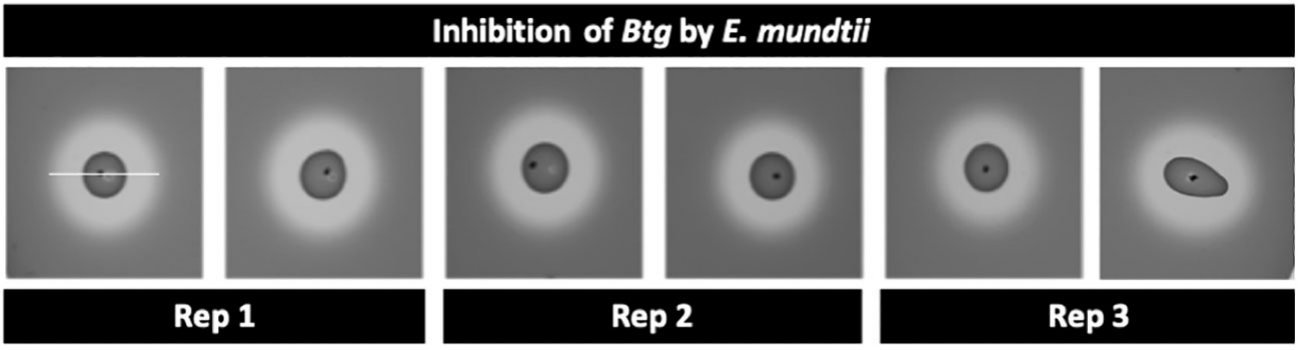
Zone of inhibition using the agar-spot-on-lawn technique between 24-h culture of *Enterococcus mundtii* (“spot”) and a 24-hour culture of *Bacillus thuringiensis* subsp. *galleriae* 69-6 (“lawn”/indicator strain); the area from edge to edge of the inhibition zone (measurement indicated by white bar) is 19 mm ± 1 mm.

The inhibition assays using the “agar-spot-on-lawn” technique (43) with *E. mundtii* against *Bacillus thuringiensis* subsp. *galleriae* 69-6 yielded inhibition zones around the spot of *E. mundtii* (**Figure 4**). This showed that the *E. mundtii* isolated from the conventional larvae gut had some antimicrobial activity against *Btg in vitro.*

### 3.6 Basal immune gene expression is stimulated by gut microbiota

**Figure 5:**
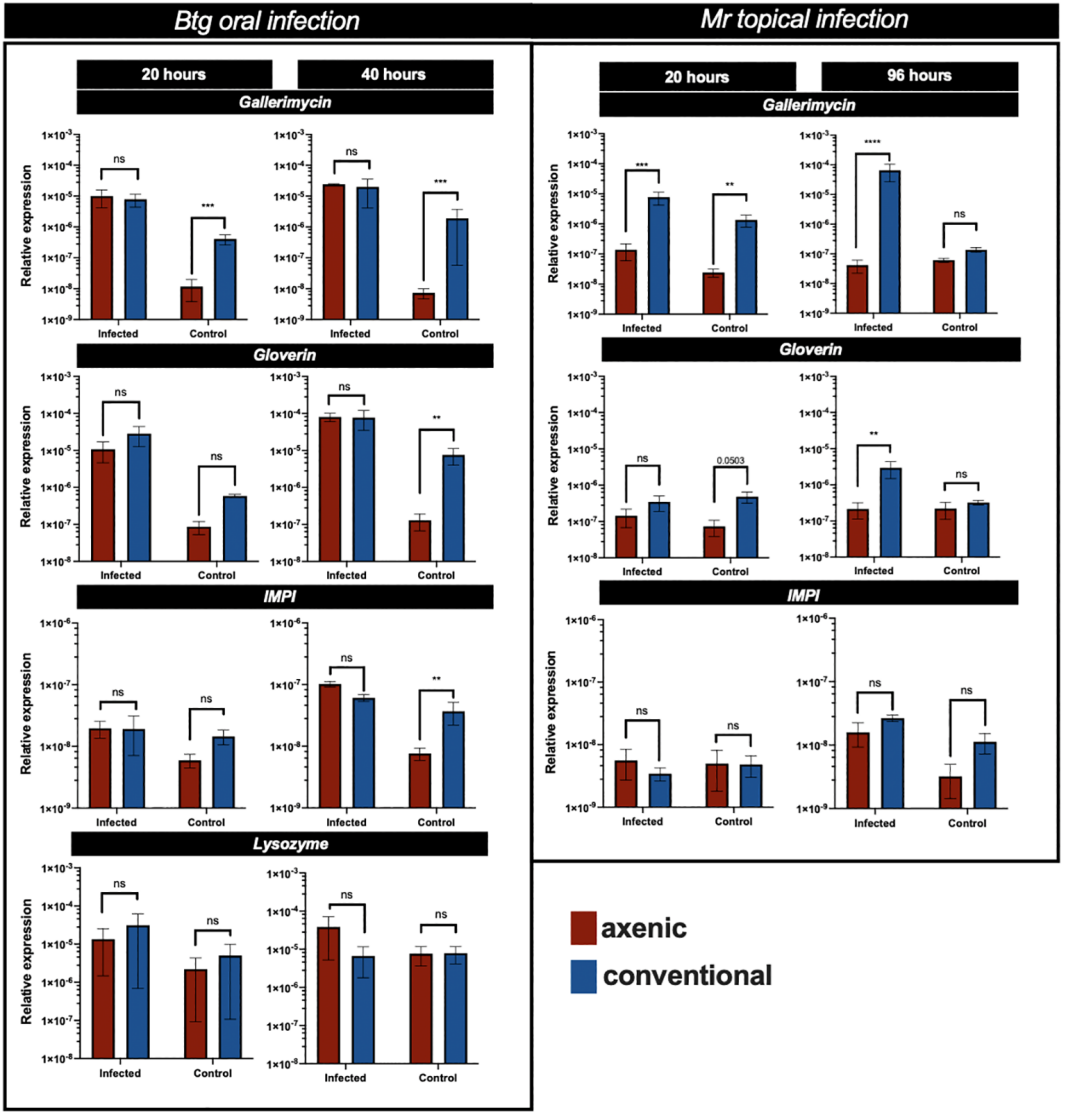
The relative expression of several immune response genes in the gut of axenic (red) and conventional larvae (blue), expressed as a ratio over 18S House keeping gene (HKG), at different time points post infection, along with respective controls. For the oral *Btg* infection and uninfected controls, relative expression is assessed at 20 h and 40 h, as for the topical *Mr* infection and uninfected controls, relative expression is assessed at 20 h and 96 h. Significance was determined by one-way ANOVA on log2-transformed values; ns (not significant) = P > 0.05; * = P ≤ 0.05; ** = P ≤ 0.01; *** = P ≤ 0.001; **** = P ≤ 0.0001.

The results of the qRT-PCR of several immune response genes revealed that strong differences were observed in the basal (uninfected control group) levels of expression of gallerimycin, gloverin, and *IMPI* between the axenic and conventional larvae (**Figure 5**). This was statistically significant for gallerimycin at 20 h (*p* = 0.0024) and 40 h (*p* ≤ 0.0001), as well as at 96 h for gloverin (*p* = 0.0048) and *IMPI* (*p* = 0.007). However, during *Btg* infection, there was a similar level of gene expression between the axenic and conventional larvae. In fact, there were no significant differences found during *Btg* infection at either 20 h or 40 h post exposure. Notably, no significant differences were observed in the relative levels of expression of lysozyme at either 20 h or 40 h between the axenic and conventional larvae, at both basal conditions as well as post-*Btg* infection.

The levels of gene expression in the gut post-topical infection of *Mr* were different from the orally infected *Btg* infection. At 20 h post infection, there was a significant difference in the expression of gallerimycin (*p* = 0.0003) between the axenic and conventional larvae, with the conventional larvae experiencing higher levels of expression (**Figure 5**). It is expected that the levels of gene expression in the gut would be lower following a topical infection than an oral infection; however, at 96 h only the conventional larvae experienced an increase in important AMP expression (gallerimycin, *p* ≤ 0.0001; gloverin, *p* = 0.0027). At basal (uninfected) conditions a significant difference was observed in gallerimycin (*p* = 0.0017), and almost significantly for gloverin (*p* = 0.0508) at 20 h post infection; however, no significant differences in the levels of gene expression were observed at 20 h for *IMPI*, and at 96 h, no genes were significantly more expressed in either the axenic or conventional larvae.

### 3.7 A global view of the fold change in gene expression from basal to infected state

**Figure 6:**
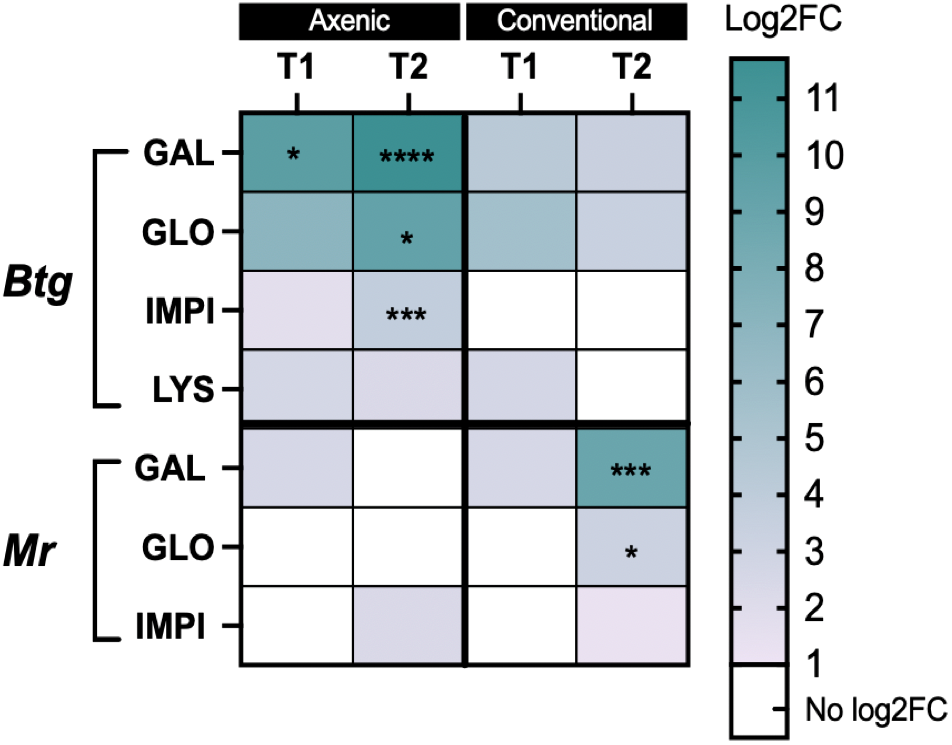
Heatmap of the log2-fold change gene expression from basal to infected conditions in the axenic and conventional larvae by either *Btg* or *Mr* at two time points post infection. The significance was determined for the difference in the fold change in expression of each gene and the time point between the conventional and axenic larvae by one-way ANOVA on log2-transformed values. * = P ≤ 0.05; ** = P ≤ 0.01; *** = P ≤ 0.001; **** = P ≤ 0.0001. T1 = 20 h post infection for both Btg and Mr, and T2 = 40 h or 96 h post infection for Btg and Mr, respectively

During *Btg* infection, the axenic larvae had a greater fold change in gene expression from an uninfected to an infected state, which was significantly different from conventional larvae at 20 h post infection for gallerimycin (*p* = 0.0226) as well as at 40 h post infection for gallerimycin (*p* ≤ 0.0001), gloverin (*p* = 0.0183), and *IMPI* (0.0004). During the *Mr* infection, the fold change in gene expression was significantly greater only in the conventional larvae gut than in the axenic group for the expression of gallerimycin (*p* = 0.0009) and gloverin (*p* = 0.025) at 96 h post infection, and no fold change in the level of expression in these important immune response genes was found in the axenic group (**Figure 6**).

### 3.8 PO activity increased post *Btg* infection but decreased post *Mr* infection

**Figure 7:**
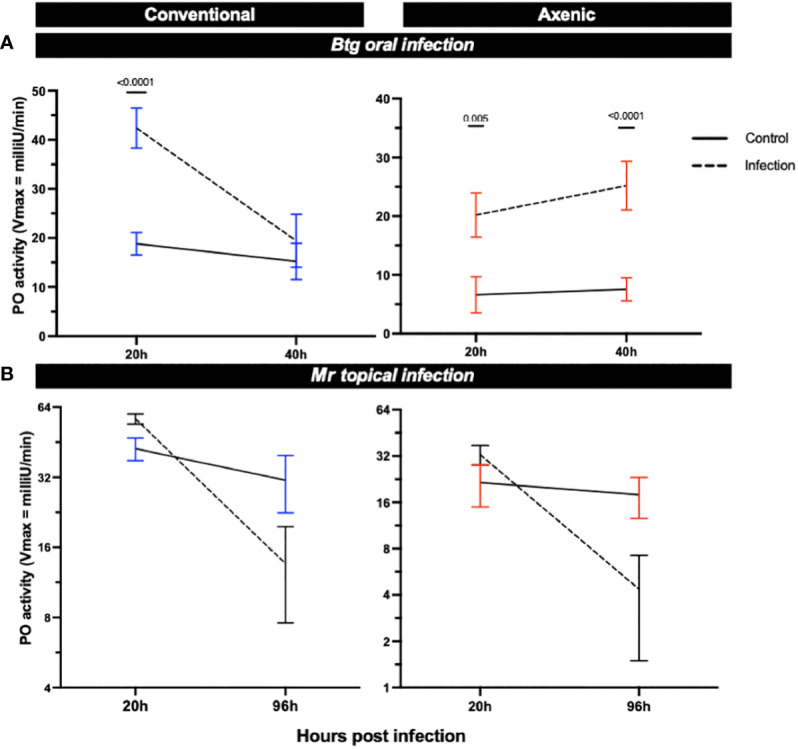
The phenoloxidase (PO) activity in the hemolymph of surviving axenic and conventional larvae infected either **(A)** orally by *Btg*, or **(B)** topically by *Mr.* Significance was determined by ANOVA–Kruskal–Wallis followed by Dunn’s multiple comparison test.

We measured the PO activity in the hemolymph of orally and topically infected larvae reared axenically or conventionally to further investigate the role of the microbiota in different immune responses in its host (**Figure 7**). The larvae, independent of microbiota status, experienced an increase in PO activity post-*Btg* infection as compared with the uninfected controls. For the *Btg* infection, at 20 h both the axenic and conventional larvae experienced significant increases in PO activity (*p* = 0.005 and *p* ≤ 0.0001 for axenic and conventional larvae, respectively). The significant increase was maintained at 40 h for the axenic larvae (*p* ≤ 0.0001), but not for the conventional larvae. For the topical *Mr* infection, a slight increase in PO activity was observed at 20 h post infection (although this was not statistically significant), followed by a decline in activity at 96 h post infection for both the axenic and conventional larvae. No significance was found at 96 h for either the conventional or axenic larvae; however, there was considerable variation in the amount of PO activity, potentially corresponding to the variation in cadaver phenotypes post death by *Mr* (**Supplementary Figure S9**), in which some larvae experienced higher levels of melanization with a complete absence of melanization in others.

### 3.9 Total free radicals (ROS/RNS) were greater in the gut of uninfected axenic larvae than in uninfected conventional larvae

**Figure 8:**
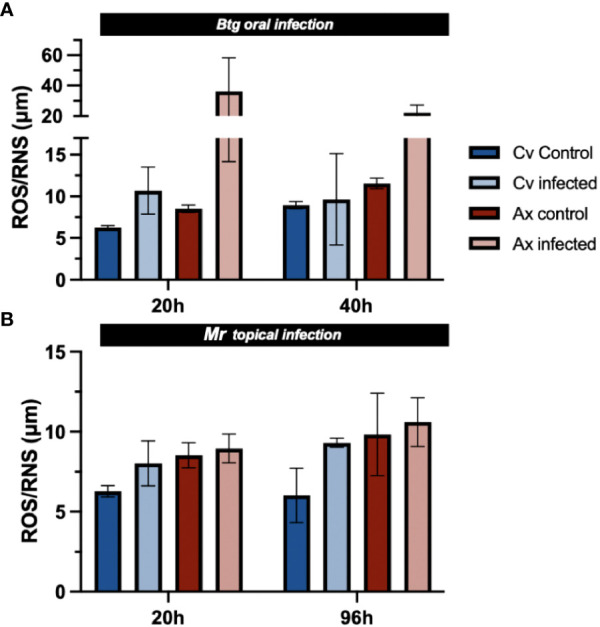
The total ROS/RNS in the gut of nine surviving larvae (three guts per sample × 3) during **(A)** *Btg* and **(B)** *Mr* infections at two time points corresponding to early and late infections. No significance was found using non-parametric one-way ANOVA.

The level of total free radicals (ROS/RNS) was slightly higher in uninfected axenic larvae than in uninfected conventional larvae (**Figures 8A, B**). During infection by *Btg*, surviving axenic larvae experienced an increase in total ROS/RNS in the gut environment at 20 h post infection (4.2× increase from the uninfected state average), as well as at 40 h post infection (1.9× increase). Post infection (1.7× increase), although not to the same level as axenic larvae. No increase in ROS/RNS was observed at 40 h post infection for the conventional group, and ROS/RNS production was not significantly induced by the topical *Mr* infection, although a slight increase for both the axenic and conventional larvae was observed during infection compared with the controls.

## 4 Discussion

Understanding the relationship between the gut microbiota and immune response is important to improve strategies for maintaining insect health. We have provided data on this relationship between the microbiota and immune response in the gut following infection by pathogens with different routes of entry into the host. We have made a comparative analysis of the immune response and growth parameters between conventional larvae and those reared in the absence of the involvement of the gut microbiota. Broad-spectrum antibiotics are often used to generate germ-free insects to gain insights into the role of the gut microbiota in host tolerance to various foreign microbes. However, this approach can have indirect detrimental effects on the host, such as interfering with protein synthesis mechanisms (46). Furthermore, the use of antibiotics in generating an axenic host may not be suitable for studies that require targeted infection of the host with bacterial strains, as they may themselves be susceptible to antibiotics (47, 48). Through the combination of UV sterilization of the egg surface coupled with rearing on a sterile diet, we achieved a successful and effective method of generating *G. mellonella* axenic larvae, as was verified using PCR and CFU counts. For certain insects, such as *Aedes aegypti* (Diptera: family) and *Apis mellifera* (Hymenoptera: family), the gut microbiota is required to complete its life cycle (49, 50), and in the Lepidoptera, the microbiota has been shown to improve the growth rate of larvae on suboptimal diets (51). Thus, we examined the effects of harboring a gut microbiota, or not, on *G. mellonella* growth and development. Our results indicate that the gut microbiota is not required for a successful completion of the life cycle in *G. mellonella*. The insect could successfully pupate and reach the adult life stage in the absence of a gut microbiota, albeit with more variation, particularly at the larval stage (**Figures 1A, B, C**). There was no difference in pupal weight, suggesting that the larvae pupate once they reach a critical weight, even if it delays their time to pupation (**Figure 1D**). Therefore, it appears that in *G. mellonella*, the resource allocation of harboring a microbiota and/or its role in regulating the immune response to maintain its homeostasis and tolerance to beneficial microbes has no visible effects on the biological parameters we have measured and does not seem to compromise other important physiological functions that would impact the insect life cycle. To investigate whether or not the gut microbiota plays a role in protecting *G. mellonella* against infection, we then used two pathogens to challenge axenic and conventional *G. mellonella* larvae via different infectious routes. The results show that when both the axenic and conventional larvae were force fed by a spore crystal preparation of *Btg* at a half-lethal dose for the conventional group, the percentage mortality was always greater for the axenic larvae, with between 90% to 100% mortality by 96 h. These results demonstrate that *G. mellonella* larvae with a gut microbiota are less susceptible to *Btg* infection than larvae with an absence of microbiota (**Figure 1E**). In contrast, for the *Mr* infection there is no significant difference in the mortality in the infected larvae when they were given a half-lethal dose, indicating that the presence of the gut microbiota does not aid in the protection from fungal pathogens entering topically (**Figure 1F**).

### 4.1 Dominance of *Enterococcus* and its role in maintaining gut microbiota composition

Our metagenomic analysis of the bacterial microbiota of our *G. mellonella* population revealed that our strain is associated with a relatively small number of bacterial taxa, dominated by a subset of *Enterococcus* species with a very high prevalence of *E. mundtii, E. gallinarum, E. casseliflavus*, and *E. inessii* (**Figure 3**). Any observed effects on the immune response were as a result of the presence of this genus of bacteria. A previous study also found only *Enterococcus* species in the *G. mellonella* gut (52); however, more recently published studies have also identified, along with *Enterococcus* but in lower abundances, *Enterobacter, Bacillus, Halomonas, Shewanella, Pseudomonas, Variovorax, Staphylococcus, Serratia*, and *Escherichia–Shigella* present in the late-larval instar of *G. mellonella* (28, 41, 53–56). With a more diverse microbiota, shifts in the dominance between bacterial groups post-pathogen infection have been observed. In particular, studies led by Dubovskiy et al. (41) and Grizanova (2022) found that *Enterobacter* (a genus of Gram-negative bacteria) maintained a minor presence in the gut community of *G. mellonella* at basal conditions, but proliferated during infection by *Btg* to overwhelm and dominate the community. Similar shifts have been observed post-fungal infection with *Beauveria bassiana* (54). These observed shifts are in contrast to what we have found, as the microbiota community remained stable without drastic changes in response to oral *Btg* or topical *Mr* infection. Indeed, the alpha- and beta-diversity analyses (**Supplementary Figures S5, S6**) showed no significant differences in the microbiota communities from uninfected control samples to the infected samples. This is interesting as the development of infections is not only restricted to the pathogen. For example, an increase in mortality due to enteric bacteria of the microbiota playing a synergistic role with pathogenic bacteria and fungi during infections has been found, increasing the mortality rates in its host (16, 47, 57). However, we did not test for absolute abundance, which may have provided some more information on the status of the microbiota during these infections. We nevertheless found that when both axenic and conventional larvae were topically infected with a half-lethal dose of *Mr*, their mortality rates were similar, indicating that the microbiota did not play a role in assisting the fungal infection. We did, however, observe an improved fitness in the conventional larvae with the oral *Btg* infection. The improved fitness could be in part due to the presence of *E. mundtii*, which was identified by 16S sequencing as well as by isolating it from the conventional larvae gut. Tang et al. (2012) previously discovered that *E. mundtii* accounted for 40% of the entire microbial gut community in *Spodoptera littoralis* (Lepidoptera: Noctuidae) larvae, and that it exhibited the ability to eliminate both other *Enterococcus* bacteria and other harmful invading bacteria that posed a significant threat to the larvae’s survival (58). As *E. mundtii* was a dominant member in the microbiota community of our *G. mellonella* population, and we observed some variation in the relative abundances of the *Enterococcus* species at different times and larval cohorts, it would be interesting to further test the competition and community dynamics between the *Enterococcus* species, to try to uncover and understand their colonization strategies.

We were driven to assess if the *E. mundtii* isolated from the conventional larva gut exhibited inhibition to *Btg*, as *E. mundtii* is known to secrete bacteriocins that have previously been shown to have broad-spectrum antimicrobial activity against Gram-negative and Gram-positive bacteria, including *Bacillus thuringiensis* (17, 43). Grau et al. (43) found that when *Tribolium castaneum* (Coleoptera: Tenebrionidae) beetle larvae were inoculated with a probiotic isolate of *E. mundtii* (or the supernatant), the larvae experienced improved survival to *Btg* but not to Gram-negative *Pseudomonas entomophila.* Furthermore, a recent study on *Hyphantria cunea* (Lepidoptera: Erebidae) found that germ-free larvae inoculated with *E. mundtii* reduced the mortality of larvae infected with *Btg* as well as a nucleopolyhedrovirus (NPV). However, germ-free larvae inoculated with *Klebsiella oxytoca* (a species of Gram-negative *Enterobacter*) were found to be significantly synergistic with the effects of both *Btg* and the NPV, resulting in increased mortality (17). Using the same zone of inhibition technique as Grau et al. (43), we indeed observed inhibition zones between the *E. mundtii* against *Btg*, confirming that this dominant microbiota member has antimicrobial activity against *Btg in vitro* (**Figure 4**). Shao et al. (59) found that a strain of *E. mundtii*, isolated from the gut of *S. littoralis*, produced a stable class IIa bacteriocin, mundticin KS, which strongly affects Gram-positive pathogens while having no or little effect on other resident gut bacteria. It would therefore be interesting to further test the antimicrobial activity of the *E. mundtii* isolate on other commensal bacteria to understand if it plays a role in the establishment and development of the microbiota community.

### 4.2 Immunostimulatory role of *Enterococcus* species

We were able to identify a faster rate of clearance of the *Btg* spores from the gut of conventional larvae (**Figure 2**), which was likely due to colonization resistance factors of the commensal gut microbiota such as space utilization, the secretion of antimicrobial inhibitors, and immune “readiness”, as found by the increased basal expression of immune-relevant genes (**Figure 5**). The conventional larvae had increased levels of expression of all three immune response genes tested (gallerimycin, gloverin, and *IMPI)*, which could result in a faster response to the *Btg* infection. Krams et al. (60) also observed an upregulation of AMP genes (including gallerimycin and gloverin) in larvae with a higher number of enterococci symbionts than in antibiotic-treated larvae. Krams et al. (60) further hypothesized that the elevated expression of AMP genes may be a prophylactic response to pathogens, which, from observations in our study of the improved response to *Btg* infection in larvae harboring the enterococci symbionts, agrees with this proposed hypothesis. However, more research is required to understand the specificity of the prophylactic response, as the mortality rates were similar during the topical fungal infection in larvae with and without a gut microbiota, even though larvae harboring the microbiota also maintained an upregulation of intestinal AMP gene expression at basal conditions. Furthermore, no significant differences were found in the fold change of gene expression from the control to the infected state at 20 h post-topical infection. This may simply suggest that at an early time point of infection, the fungi, entering its host topically, may not yet strongly induce the immune response in the gut (**Figure 6**), and although the focus of this study was on the interaction of the microbiota community and localized gut immune responses during infection, the gene expression in the body fat may have been an important tissue to evaluate. We did, however, measure the PO activity in the hemolymph (**Figure 7**), which would likely be a faster indicator of a topical infection, and indeed a slight increase in PO activity in the hemolymph was observed in both axenic and conventional larvae at 20 h post infection, followed by a decrease in activity by 96 h. The initial increase may be a response to the presence of the germinating conidia on the cuticle (**Supplementary Figure S10**); furthermore, an increase in PO activity is commonly reported in the initial stages of mycoses in both *Beauveria bassiana* and *Metarhizium* species, followed by a decrease at acute stages (61–64). Entomopathogenic fungi possess the ability to inhibit melanization and other immune defenses through the production of metabolites that interfere with anti-microbial processes (65, 66). We also observed a large variation in the melanization of cadavers, post-topical *Mr* infection, in which some had completely melanized while others died without experiencing any melanization. This observation was seen in both conventional and axenic larvae. It is worth noting that at 96 h, the immune-stimulatory effect on gene expression by the microbiota in conventional larvae was diminished, as no significant differences in basal immune expression were detected between the conventional and axenic larvae. This absence of immune stimulation in conventional larvae at 96 h post *Mr* infection could be attributed to the age of the larvae, which were close to pupation. A decrease in midgut AMPs close to pupation has also previously been observed in prepupal *G. mellonella* larvae (67). Therefore, the diminished immune stimulation by 96 h, and the purported lack of participation in the fungal infection by the microbiota (due to no significant changes in the relative abundance or significant differences in alpha- and beta-diversity metrics between the control and infected larvae), resulted in similar mortality rates between the axenic and conventional larvae.

We also examined the inducibility of ROS/RNS in the gut environment during infections (**Figure 8**). The axenic larvae, more susceptible to the dose of *Btg* than the conventional larvae, experienced greater oxidative stress in the gut environment. An excessive production of ROS/RNS can contribute to cell structure damage, likely causing significant cell lysis and aiding pore formation in the gut, contributing to mortality (68, 69). In general, there were no significant changes to the level of gut ROS/RNS between all *Mr* infected and uninfected larvae. The slightly higher levels of ROS/RNS in the gut of all uninfected axenic larvae could suggest that the *Enterococcus*-dominated microbiota may potentially produce some antioxidants and enzymes that help scavenge ROS in normal conditions, thereby maintaining the gut environment’s oxidative balance, which may have also played a role in attenuating oxidative stress post-*Btg* infection in the conventional larvae. The antioxidant properties in *Enterococcus* species have been increasingly studied, as these bacteria have exhibited the potential to inhibit pathogenic microbes as well as reduce oxidative spoilage in foods and feed (70–72).

### 4.3 Implications and future directions

In addition, we recognize that the immune response is modulated by additional genes than those tested in this study, as well as molecules not traditionally considered “immune genes” but which are also important for the immune response (41, 73). Therefore, a wider view using a larger transcriptomic approach may provide a more comprehensive understanding of the immune system stimulation by the gut microbiota both before and during infections. The dynamics of the immune stimulation of the microbiota under different stresses and environmental conditions also deserve further attention, including how the microbiota could influence more systematic immune responses. Understanding the insect gut microbiota–pathogen interactions offers potential benefits for future insect control and insect health strategies through targeted manipulation or exploitation of these interactions. For example, by introducing beneficial microbes into insects or their environments, it may be possible to develop probiotics or prebiotics that can modulate the gut microbiota composition and function to enhance host resistance or tolerance to pathogens. In particular, we plan to look into the role and the effect of the isolated *E. mundtii* strain on the immune response of *G. mellonella* larvae by assessing their ability to restore the resistance to *Btg* after repopulating axenic larvae. By generating gnotobiotic *G. mellonella* larvae, we could determine if the immune response can be improved by a single bacterial isolate or if a synergistic effect between the multiple bacterial *Enterococcus* species is needed.

### 4.4 Conclusion

In summary, our study revealed that a restricted number of bacterial strains in the larval gut significantly enhanced their survival rate following *Btg* infection but had a neutral role in the pathogenesis of topically inoculated *Mr*, in the economically important laboratory infection model, *Galleria mellonella.* Moreover, our results demonstrated a substantial impact of these *Enterococcus* species on the basal level of expression of immune-related genes, underscoring the crucial role of the gut microbiota in the immune response of *G. mellonella*. These findings suggest that the gut microbiota of *G. mellonella* can serve as a protective mechanism against pathogenic infections caused by the bacterial pathogen *Btg*. This protection may be attributed to various factors, including, as discussed, the modulation or enhancement of the host immune system’s basal level of immune activation or “readiness.” In addition, the gut microbiota, dominated by *Enterococcus* species, might contribute to this defense through the production of antimicrobial compounds or by competing with pathogens for vital resources such as nutrients or space. Collectively, these mechanisms highlight the important role of the gut microbiota in safeguarding *G. mellonella* against certain pathogenic threats. This work adds value to the readily discussed role of the microbiota, reinforcing the reality that its role depends largely on the species composition of the microbiota, the host rearing conditions, and the type of pathogen. Moreover, some studies have shown that insects have the ability to mount specific and enhanced immune responses following prior exposure to a pathogen (74). Whether or not some members of the resident insect gut microbial communities can also function as “immune priming agents” capable of inducing the expression of genes involved in the recognition and elimination of pathogens is not yet clearly established and remains to be explored. Understanding these relationships will provide insight for new strategies on how to maintain insect health when they meet diseases from multiple and diverse pathogens.

## Data availability statement

The original contributions presented in the study are included in the article/**Supplementary Material**. Further inquiries can be directed to the corresponding author.

## Ethics statement

Ethical review and approval was not required for the study on animals in accordance with the local legislation and institutional requirements.

## Author contributions

JU: Conceptualization; Data curation; Formal analysis; Investigation; Methodology; Visualization; Writing — original draft; and Writing — review and editing. AR: Conceptualization; Data curation; Methodology; and Writing — review and editing. CN-L: Conceptualization; Funding acquisition; Methodology; Project administration; Resources; Supervision; Visualization; and Writing — review and editing. AJ: Conceptualization; Funding acquisition; Methodology; Project administration; Resources; Supervision; Visualization; and Writing — review and editing. VS-B: Conceptualization; Funding acquisition; Methodology; Project administration; Resources; Supervision; Validation; Visualization; and Writing — review and editing.
