# Supplementary material for: The immunostimulatory role of an Enterococcus-dominated gut microbiota in host protection against bacterial and fungal pathogens in Galleria mellonella larvae
Source: Front Insect Sci. 2023 Oct 26;3:1260333. doi: 10.3389/finsc.2023.1260333 (PMC10926436; doi:10.3389/finsc.2023.1260333)
Supplement: Supplementary file 1 [file DataSheet_1.docx]

Supplementary Material

Jennifer Upfold^1,2*^, Agnès Rejasse^1^, Christina Nielsen-Leroux^1^, Annette Bruun-Jensen^2^, Vincent Sanchis^1^

^1^ Université Paris-Saclay, INRAE, AgroParisTech, Micalis Institute, 78350, Jouy-en-Josas, France

^2^Department of Plant and Environmental Sciences, University of Copenhagen, Thorvaldsensvej 40, 1871, Frederiksberg, Denmark

Address for correspondence:

*** Correspondence:**Jennifer Upfold
jennifer.upfold@inrae.fr

**Supplementary Materials and Methods:**

#### Microbiota: Bioinformatics

The following modifications were made to the default functions proposed by the DADA2 workflow package. After inspecting the quality profiles of the forward and reverse reads, trimming was performed at 250 and 200 reads for forward and reverse reads, respectively (where the quality score started to drop below 30). To learn the error rates, the number of samples, bases and reads was increased with the arguments ‘nbases = 1e + 09’ and randomize = TRUE.’ The dereplication, sample inference and merging steps were then performed as proposed by the workflow. Lastly, chimeric sequences were removed. Taxonomic affiliations were performed to genera level, and species level where possible, using the SILVA reference database, version 132. To further identify to species level, a BLAST search of the resulting ASVs against the NCBI rRNA/ITS database <https://www.ncbi.nlm.nih.gov/> was conducted. Further details of the BLAST search are recorded in supplementary Table S4.

#### Statistical analyses

Analyses were performed using the R software, using the ‘phyloseq’ (v.1.26.1) and ‘microbiome’ packages (Lahti *et al.* 2012; McMurdie & Holmes 2013). Sequence taxonomy, sample metadata and ASVs were combined into a phyloseq object for further analysis. Samples were firstly plotted to identify any outliers in the sequencing depth, followed by rarefying the data using the “ggrare” function by using the “rarefy_even_depth” function and setting “set.seed(400)” with a sample size of 35000 (Suppl. Fig. S11). The relative abundance of the taxa was calculated at the genus (Suppl. Fig. S6) and species level (Fig. 3), as a percentage of total read count. Alpha diversity (α-diversity) community differences within the samples was estimated using the Simpson’s index which considers both the number of species present as well as the relative abundances of each species (suppl. Fig. S5). To visualize the Beta (β-diversity) community differences between the samples, a PCoA using jaccard distances was used. Jaccard distances was used as it only analyses the presence/ absence whereas bray-curtis distances accounts for the presence and absence of bacteria, as well as their relative abundance within a sample (Paddock et al., 2021). As the relative abundances are influence by introducing *Btg* into the community, jaccard distance is used. To further investigate the beta community on the microbiota community, all ASVs assigned to *Bacillus thuringiensis* were subsetted and discarded using the “subset_taxa” function on Phyloseq. Now the impact of *Btg* on the rest of the community can be assessed without the influence of the introduced *Btg* affecting the results. As there is low diversity in the community, just the presence of the introduced *Btg* separates the infected samples into their own group (Fig. S8A and 8B). However, in order to analyze the beta community in the rest of the community, these *Btg* reads need to be subsetted and removed, and then the PCoA was reconstructed. A PERMANOVA test, based on 999 permutations and significance at <0.05 was carried out using the ‘vegan’ (v.2.6.4) package with the adonis function (Suppl. Table S5).

#### Isolating *E. mundtii* from the conventional larva gut

To test the efficacy of one symbiotic strain on host resistance to pathogens, as compared to larvae harboring a complex microbiota and those that are sterile, we isolated *Enterococcus mundtii* from conventional larvae (Suppl. Fig. S3)*.* To isolate the bacteria, final instar larvae were chilled on ice before being surface sterilized with 70% EtOH. Three guts were dissected, pooled and homogenized in sterile H_2_O before plating the contents on to Brain-Heart Infusion (BHI) agar media. The plates were incubated for 72 hours at 30°C, before being inspected for colonies with different size, shape and colour morphologies. The majority of the colonies growing were uniform in their morphologies, and from microscope evaluation it was suspected that the colonies were *E. mundtii.* A colony was re-streaked to obtain a pure colony. For molecular verification, the isolate was cultured in liquid BHi for 24 hours. Following this, DNA was extracted and sequenced using the V3-V4 region of the 16S rRNA gene sequence and positively identified as *E. mundtii.*

Supplementary Figures:

Axenic verification:

Verification of the axenic status of the larvae is very necessary prior to further experiments. The verification is done using three techniques (i) plating larval homogenates or the dissected gut on BHiA medium; (ii) using PCR to detect amplification of the 16S rRNA gene; (iii) illumina sequencing. These three methods used in conjunction are important as the culture method is straightforward but is not always able to detect all microbes, such as those that are anaerobic or cannot be cultured, and PCR tests will detect all microbes however nucleic acid contamination can cause false positives and amplification thresholds do not completely exclude false negatives (Fig S1).


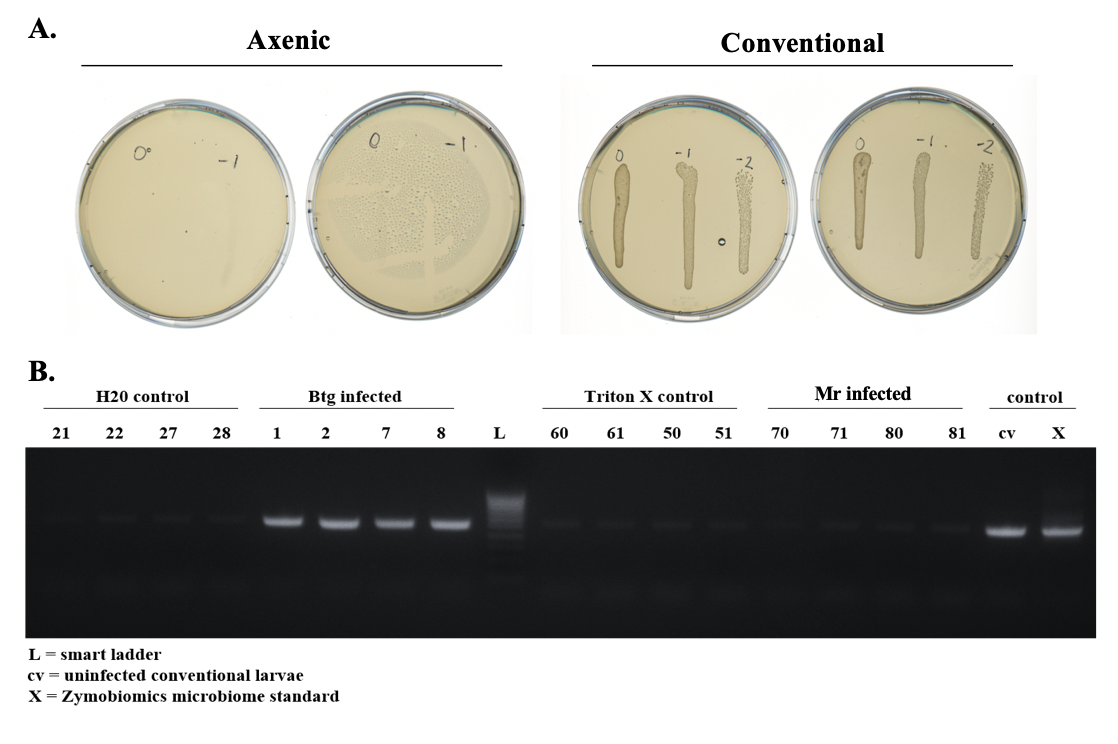


##
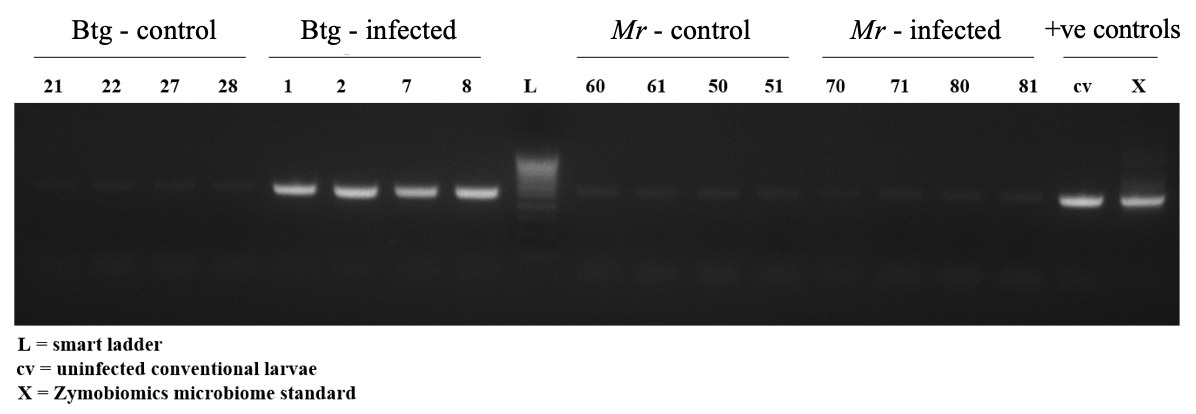


**Supplementary Figure S1. A.** Homogenized dissected gut samples, plated on BHi agar to monitor cultivable bacteria. No cultivable bacteria grew from the axenic gut (left), as compared to conventional (right). **B**. PCR amplification of the v3-v4 region of the bacterial 16S rRNA gene, amplified from axenic gut samples with bands present in the *Btg* infected larvae. Positive controls consisted of an uninfected conventional gut sample (CV) and a Zymobiomics microbiome standard (X).

**
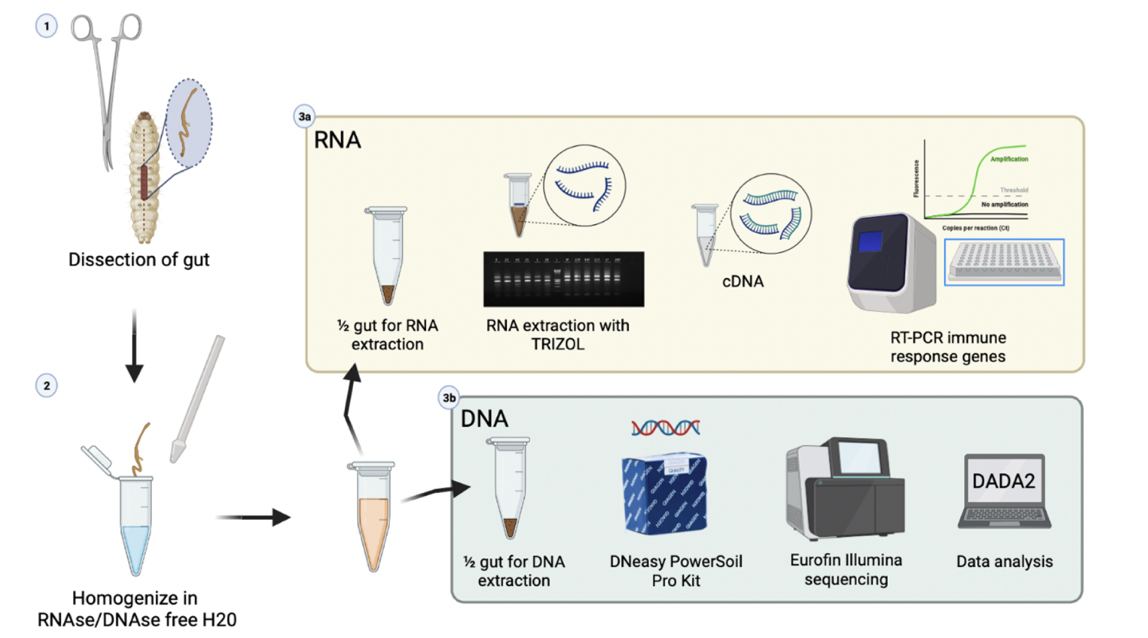
**

**Supplementary Figure S2.** schematic diagram of the dual-action analysis of the gut immune gene expression and microbiota community analysis, starting with the dissection of the gut from surviving surface sterilized larvae, then splitting the homogenized gut sample in two, one for RNA extraction with qRT-PCR and the other for DNA extraction with 16S rRNA sequencing. Each sample is comprised of half a gut from two individual larvae.


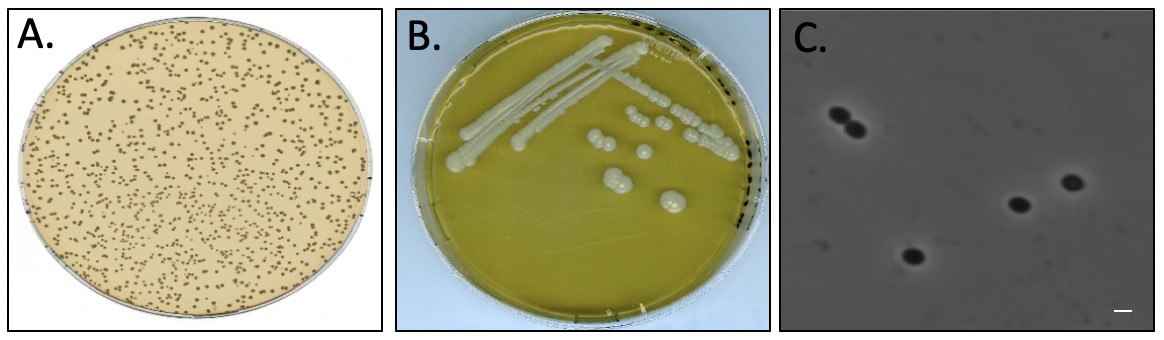


**Supplementary Figure S3.** We were able to isolate and identify the commonly found lepidopteran lab-reared symbiont *Enterococcus mundtii.* **A)** Cultivable bacteria isolated from the conventional larvae by dissecting the gut of three larvae reared on a natural diet of pollen and beeswax, and homogenizing in distilled H_2_O. **B)** Unique colonies were identified and streaked, the resulting isolate was then confirmed to be *E. mundtii* by **C)** microscopy and sequencing of V3-V4 region of the 16S gene.

**
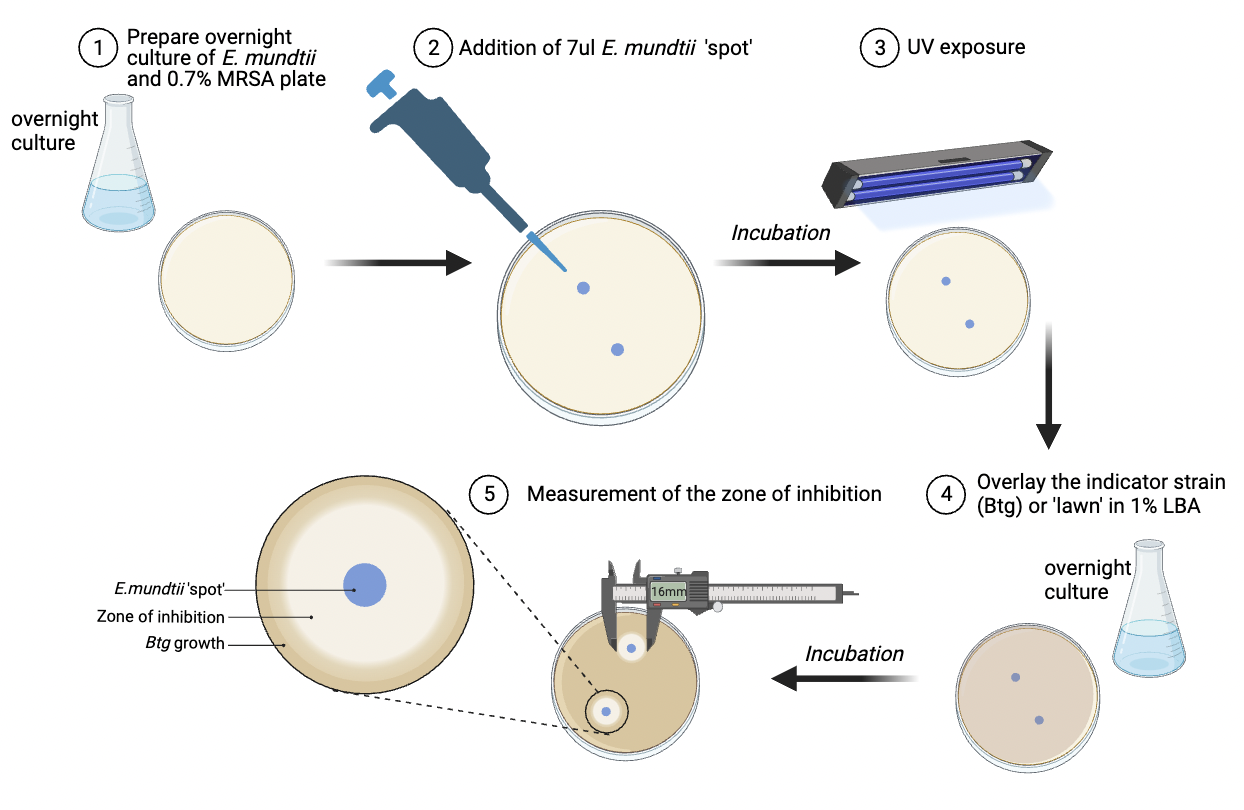
Supplementary Figure S4.** Schematic diagram of the workflow incorporating the agar-spot-on lawn technique (Schillinger and Lucke, 1989) and UV exposure to analyze the inhibitory effect of *E. mundtii* bacteriocins on *Btg in vitro.*


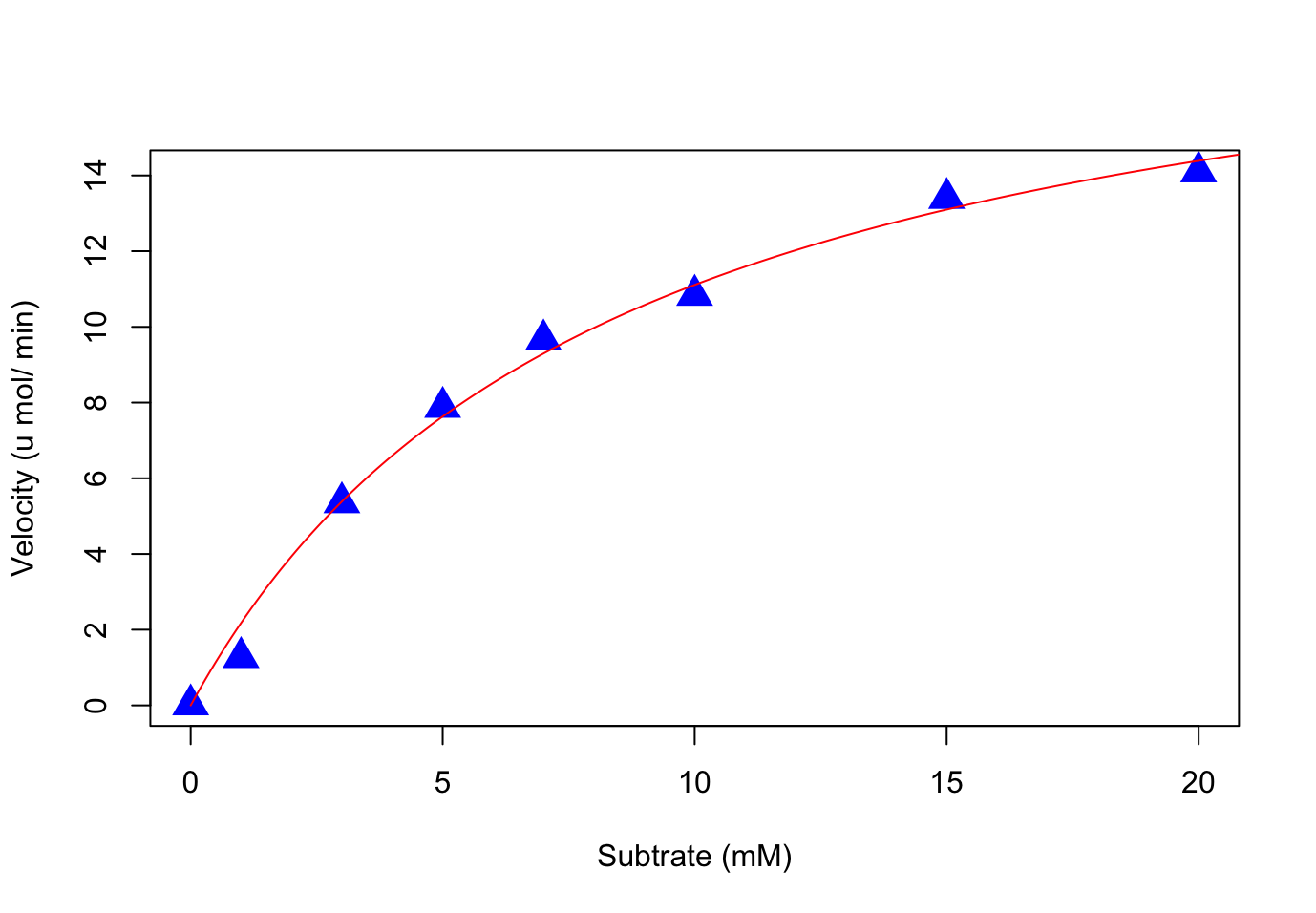


**Supplementary Figure S5.** Calibration of L-DOPA concentration for measuring phenoloxidase in the hemolymph of 250-300mg *Galleria mellonella* larvae. The phenoloxidase in the hemolymph was found to conform to the Michaelis-Menten equation with a Michaelis constant (Km) of 8,37 mM, giving an optimal velocity (Vmax, equal to 2 x Km) of 16,6mM L-DOPA.


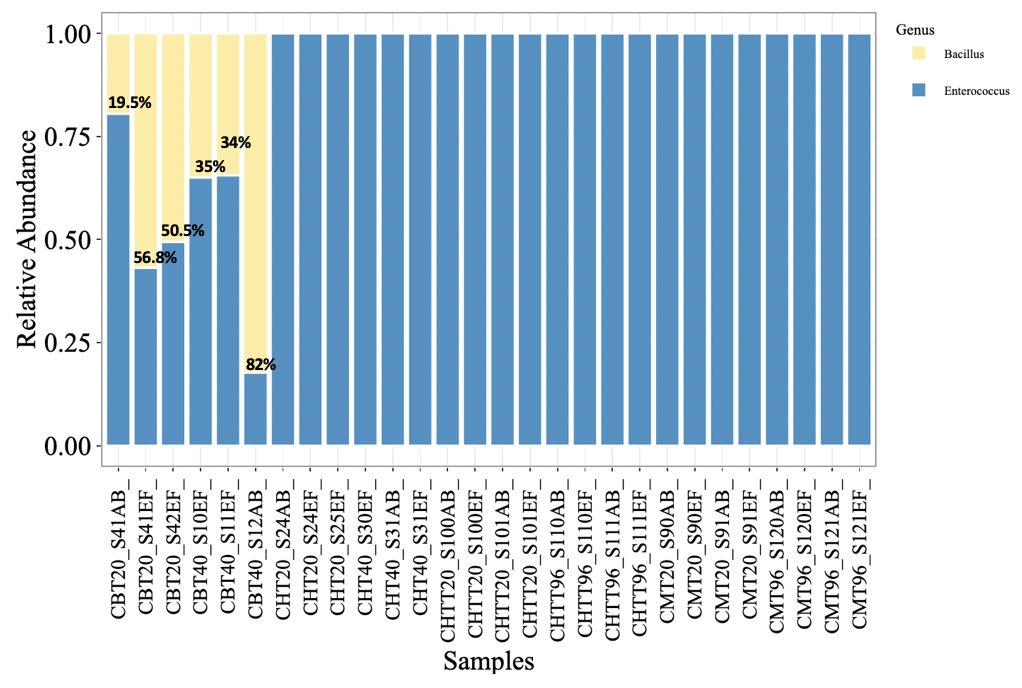


**Supplementary Figure S6.** Bar plots of the relative abundance of the most abundant genus (>1%) with the percentage of Bacillus above each bar

**Supplementary Figure S7.** The Observed and Shannon indices for Alpha community analysis displaying the means and standard deviation. The observed value is a display of the unique ASVs in a sample (post rarefied and filtered at 1/1000), whereas the Simpson index displays the diversity of those unique ASVs, where 0 would be all ASVs belong to one species, and where 1 would be all ASVs belong to different species.


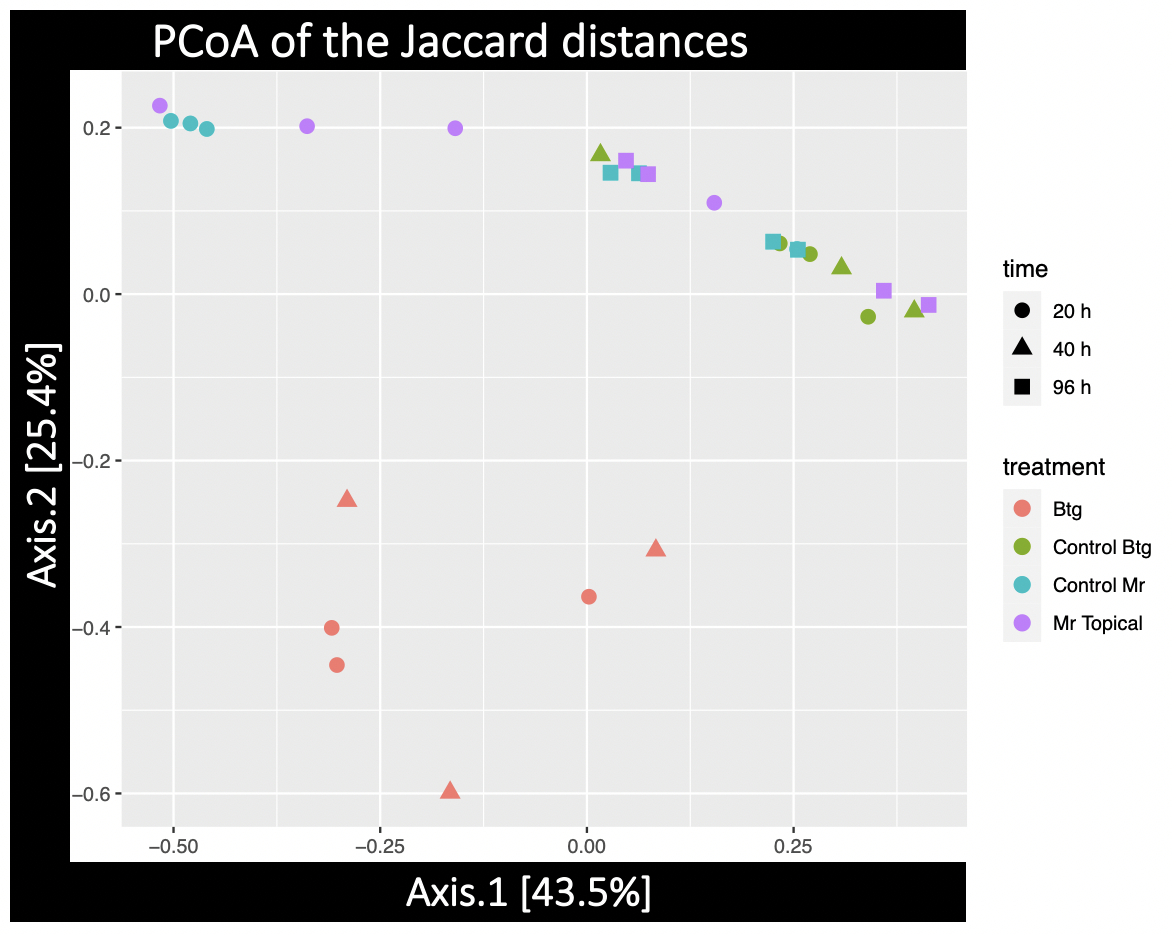


**A.**

####
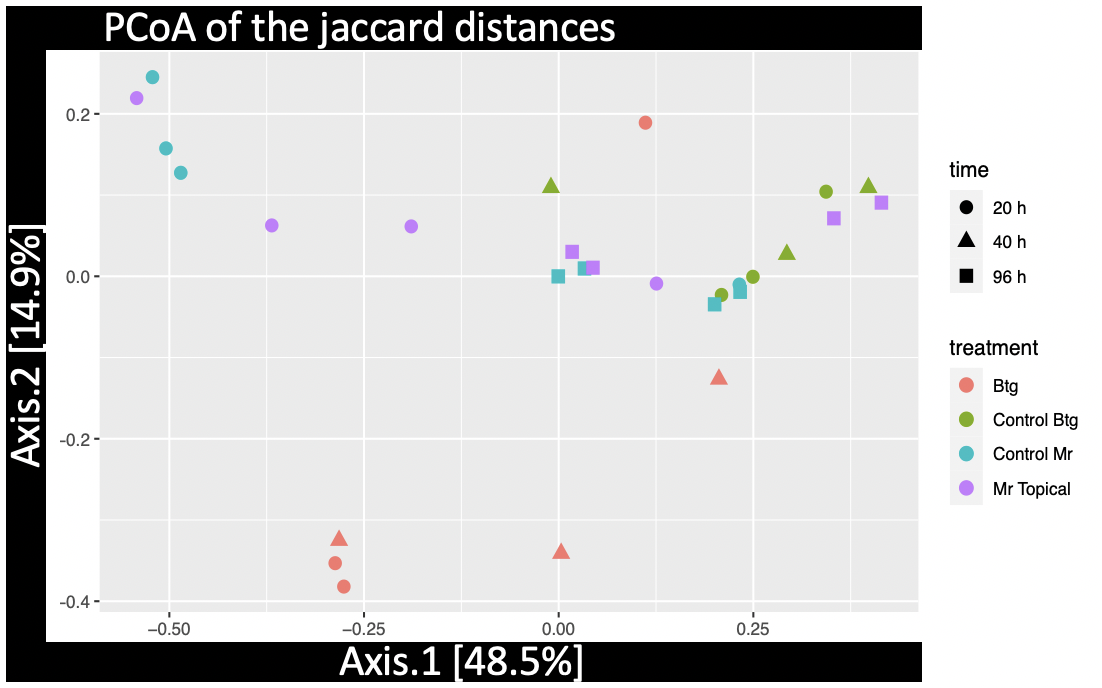


**B.**

**Supplementary Figure S8. A.** PCoA of the jaccard distances of all samples, before *Bacillus thuringiensis* ASVs were subsetted, showing the separation of the *Btg* infected samples. **B.** PCoA of the jaccard distances of all samples with the *Btg* reads subsetted and removed


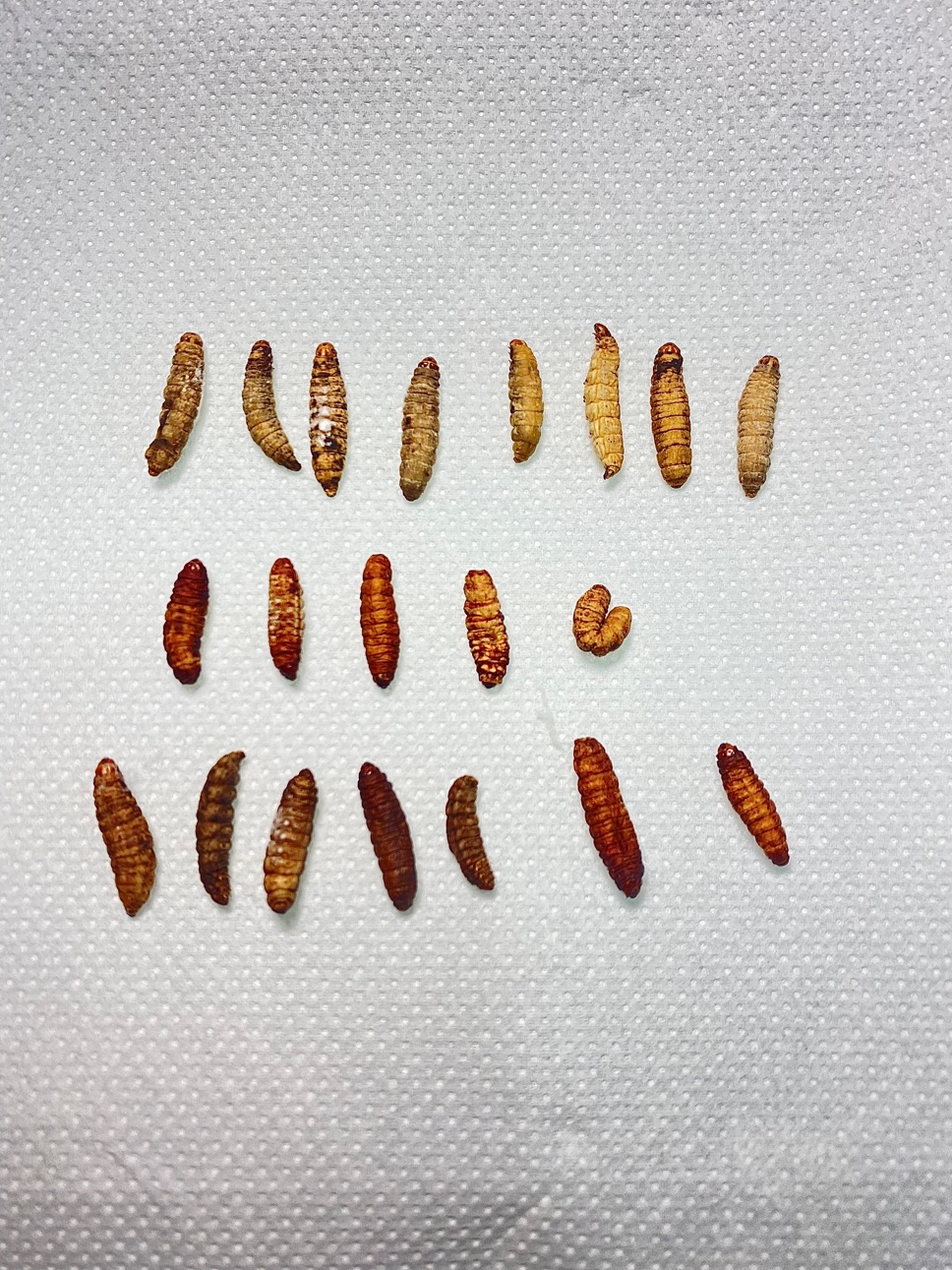


**Supplementary Figure S9.** Phenotypes of cadavers post-death by *Metarhizium robertsii KVL 00-89,* showing the spectrum of non-melanized to melanic cadaver morphs. All cadavers are mummified and stiff, unlike the softened cadavers from *Btg* infected larvae. Larvae were further placed on dampened filter paper to monitor hyphal growth. This difference in melanization during the fungal infection may be reflected in the results of the PO activity whereby the vmax values are highly variable in the 96h larvae.


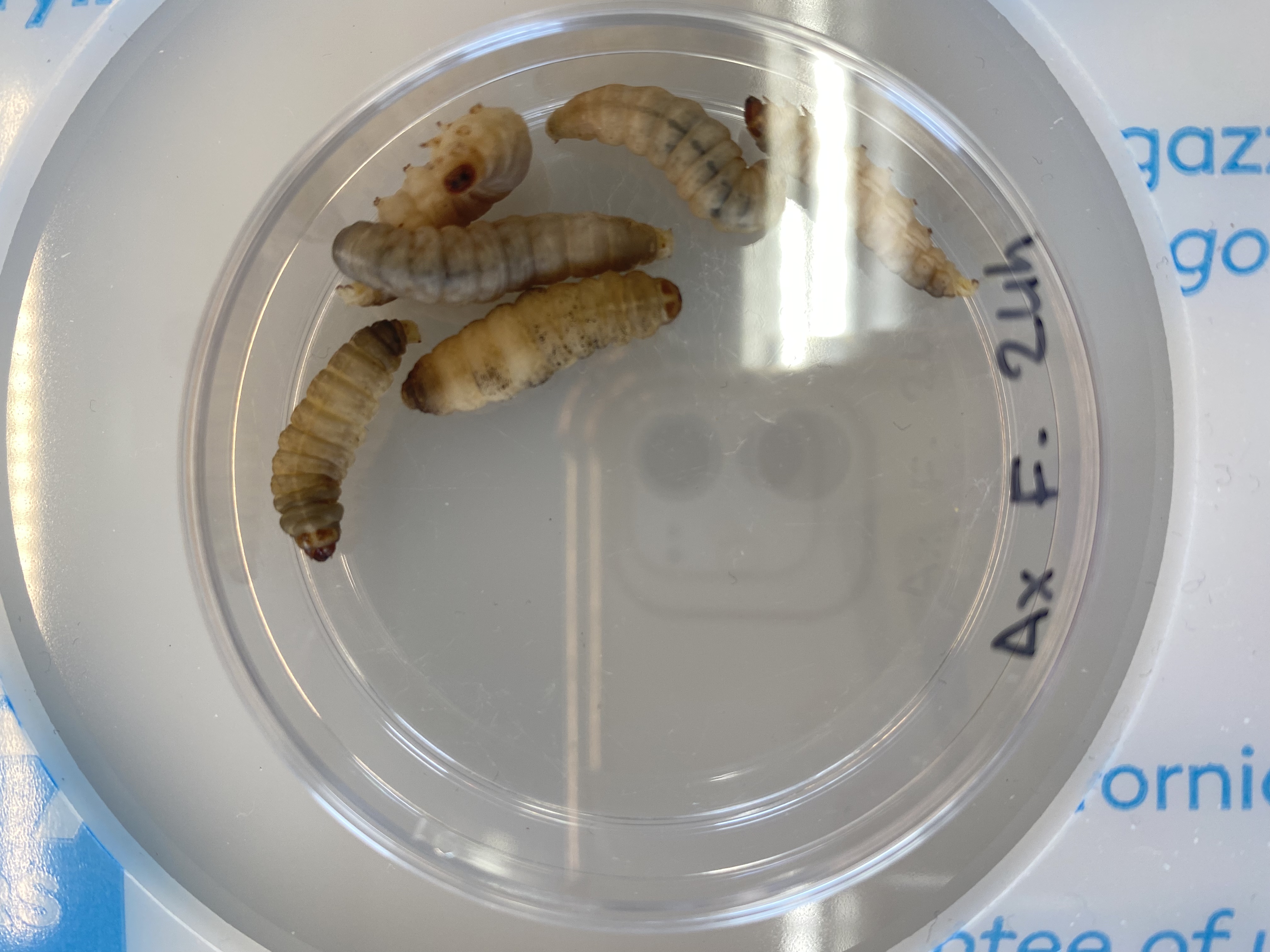

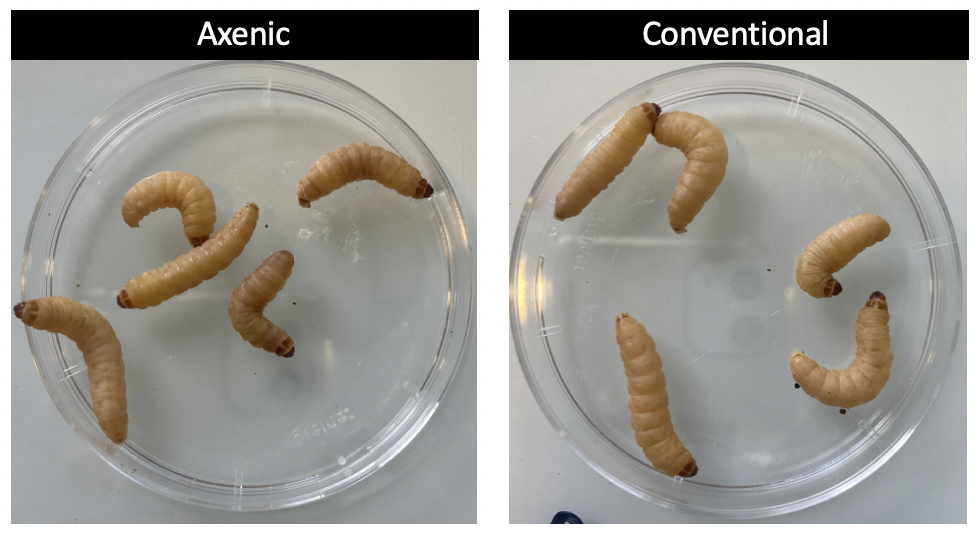


Supplementary Figure S10. Infected larvae (left) with melanized spots indicating the cuticle degradation by the Metarhizium robertsii, as compared to a healthy, uninfected larvae (right).

**Supplementary Figure S11.** The rarefaction curves for all samples using a sample size of 35000 and ‘rngseed = 400’ to ensure all ASVs were detected.

**Supplementary Tables:**

**Supplementary Table S1.** The artificial diet, prepared and autoclaved for the biological parameters assay (Mohamed & Amro, 2022)

| **Ingredient:** | **Composition of diet:** |
| --- | --- |
| liquid honey | 13,5 g |
| glycerin | 10,8 g |
| dried beer yeast | 2,7 g |
| wheat flour | 6,75 g |
| skim milk powder | 5,45 g |
| polenta | 10,8 g |
| Sterile water | 4% of total volume |
|  |  |

**Supplementary Table S2.**  Immune response genes targeting the gut tissue, used in this study

| **Primer name** | **Description** | **Immune pathway / process** | **Forward** | | **Reverse** | | **Reference** |
| --- | --- | --- | --- | --- | --- | --- | --- |
| **18S rRNA** | HKG | - | CACATCCAAGGAAGGCAG | AGTGTACTCATTCCGATTACGA | | Polenogova et al 2019, Lange et al 2018 | |
| **EF1: Elongation factor 1-alphs (Ef-1a)** | HKG | - | AACCTCCTTACAGTGAATCC | ATGTTATCTCCGTGCCAG | | Polenogova 2019 | |
| **Gallerimycin** | AMP | toll | GAAGTCTACAGAATCACACGA | ATCGAAGACATTGACATCCA | | Dubovskiy 2016, Kryukov 2020, Pereira 2018, Tsai 2016, Polenogova 2019 | |
| **Gloverin-like protein** | AMP | toll | AGATGCACGGTCCTACAG | GATCGTAGGTGCCTTGTG | | Dubovskiy 2016, Kryukov 2020, Pereira 2018, Tsai 2016, Polenogova 2019 | |
| **IMPI** | Inducible metalloproteinase inhibitor | Immune response | TAGTAAGCAGTAGCATAGTCC | GCCATCTTCACAGTAGCA | | Polenogova 2019, Lange et al 2018 | |
| **Lysozyme** |  | Immune response | GGACTGGTCCGAGCACTTAG | CGCATTTAGAGGCAACCGTG | | Lange et al 2018 | |

Supplementary Table S3. Immune response genes investigated in the study

| **Gene** | **Literature** |
| --- | --- |
| Gallerimycin | Is reportedly active against filamentous fungi but not against bacteria or yeast (1) however, its expression is strongly induced by a bacterial infection in *G. mellonella,* as observed by infection of *Photorhabdus luminescens* (Gram-negative bacterium) (2) as well as infections by *Bacillus thuringiensis* (3,4,5). |
| Gloverin | Gloverins from different lepidopteran species shows broad-spectrum activity against *E. coli,* Gram-positive bacteria, fungi and even some viruses (3,4,6,7,8,9,10) |
| IMPI | The insect metalloproteinase inhibitor, an enzyme that can degrade pathogen metalloproteinases helping to prevent or limit pathogen colonization in the host (11) |
| Lysozyme | Lysozymes, along with their digestive function in the gut, possess defense properties. It is one of the first lines of the humoral defense which creates a hostile environment for intruding microbes (11). |

1. (Schuhmann et al., 2003); 2. (Wu et al., 2016); 3. (Dubovskiy et al., 2016); 4. (Wu et al., 2022); 5. (Taszłow et al., 2017); 6. (Axén et al., 1997); 7. (Lundström et al., 2002); 8. (Hwang & Kim, 2011); 9. (Moreno-Habel et al., 2012); 10. (Xu et al., 2012); 11. (Wojda, 2017)

**Supplementary Table S4.** The result of the NCBI BLAST search of the resulting ASVs from the conventional larvae gut microbiota, generated by Illumina miseq and DADA2

| **bp length** | **NCBI BLAST** | **query cover** | **per. Ident.** |
| --- | --- | --- | --- |
| 427 | *Enterococcus mundtii* | 100 | 100 |
| 427 | *Enterococcus mundtii* | 100 | 99,77 |
| 428 | *Enterococcus gallinarum* | 100 | 100 |
| 428 | *Enterococcus gallinarum* | 100 | 100 |
| 428 | *Enterococcus gallinarum* | 100 | 99,77 |
| 428 | *Enterococcus gallinarum* | 100 | 99,77 |
| 428 | *Enterococcus gallinarum* | 100 | 99,53 |
| 428 | *Enterococcus casseliflavus* | 100 | 100 |
| 428 | *Enterococcus innesii* | 100 | 99,77 |
| 428 | *Enterococcus innesii* | 100 | 99,77 |

**Supplementary Table S5.** The results of the PERMANOVA to test the signifance of the Beta community between samples, after subsetting and discarding *Bacillus thuringiensis* reads. No significance is observed in the beta community between infected samples, infected vs their controls, as well as between control samples at different time points.

| **Group comparisons** | **F. model** | **R^2^** | **P. value** |
| --- | --- | --- | --- |
| **Infected t_1_ vs Infected t_2_** | | | |
| *Btg* 20 h vs *Btg* 40 h | 1.296 | 0.134 | 0.9 |
| *Mr* Topical 20 h vs *Mr* Topical 96 h | 3.8 | 0.388 | 0.088 |
| **Infected vs control** | | | |
| *Btg* 20 h vs Control *Btg* 20 h | 2.578 | 0.392 | 0.200 |
| *Btg* 40 h vs Control *Btg* 40 h | 1.393 | 0.218 | 0.1 |
| *Mr* Topical 20 h vs Control *Mr* 20 h | 0.469 | 0.072 | 0.634 |
| *Mr* Topical 96 h vs Control *Mr* 96 h | 0.757 | 0.112 | 0.446 |
| **Control t_1_ vs Control t_2_** | | | |
| Control *Btg* 20 h vs Control *Btg* 40 h | 0.315 | 0.073 | 0.983 |
| Control *Mr* 20 h vs Control *Mr* 96 h | 4.595 | 0.434 | 0.109 |

t_1_  = time point one (i.e. 20h) ; t_2_ = time point two (i.e. 40h or 96h)
